# First description of the male genitalia in a short-tailed whipscorpion (Arachnida: Schizomida), description of the female, and comments on pygidial glands and cuticular ultrastructure of *Surazomus algodoal* Ruiz & Valente, 2017

**DOI:** 10.1371/journal.pone.0289370

**Published:** 2023-08-08

**Authors:** Gustavo R. S. Ruiz, Roberta M. Valente

**Affiliations:** Instituto de Ciências Biológicas, Universidade Federal do Pará, Belém, Pará, Brazil; East Carolina University, UNITED STATES

## Abstract

The male genitalia of *Surazomus algodoal* Ruiz & Valente, 2017 are described. Since this is the first attempt to describe male genitalia in the schizomids, we propose new terminology to describe the sclerites of the male genital chamber in the order. We believe that the male genitalia may provide a large set of characters for taxonomic research in schizomids, as the female genitalia have proven to do. The setae and other cuticular structures were investigated using light and scanning electron microscopy (SEM). The female of *S*. *algodoal* is described for the first time, including the genitalia, and we highlight the sexual dimorphism present in the species. We also present descriptions of: 1) microtrichia, with respect to their presence/length/distribution, 2) cuticular sensilla, glandular openings and other cuticular structures, proposing new terminology, 3) setae of the pedipalpal trochanter and tarsus. Some changes on the terminology of setae of pedipalpal tibia are also given. These advances may be useful for description in other schizomids. The pygidial glands, mostly ignored in modern papers, were assessed and commented upon.

## Introduction

Short-tailed whipscorpions (order Schizomida) are a small group of arachnids (about 380 species [1]) with minute bodies (generally less than 5 mm in length; [2]) and evident sexual dimorphism in the pygidial flagellum, which is modified in the male for use in the mating march (see Ruiz & Valente [3]; Kallal *et al*. [4]). Given their small, poorly sclerotized bodies, they are restricted to highly humid environments, and may live in litter, under rocks, logs or in caves [2, 5–12]. Presently the species are distributed in two families, Protoschizomidae Rowland, 1975 (only 16 species, all in North America) and Hubbardiidae Cook, 1899, the latter with two subfamilies, Megaschizominae Rowland, 1973 (two African species) and Hubbardiinae Cook, 1899 (the remaining species, worldwide) [1].

The first species of the order was described only by the end of the XIX century [13], but almost immediately, the morphology of this interesting group received good descriptions [14–17]. Hansen & Sørensen [18] described in detail the bodies of both sexes of a series of species, including lyriform organs on the chelicera, pedipalp and legs. They also prepared drawings of the sclerites covering the prosoma, chelicera with different setae, legs with trichobothria (“tactile hairs”), booklungs, opening of the pygidial (“odoriferous”) gland and pygidial flagellum of males, females and immatures with setae. They also presented a pair of drawings of a spermatophore attached to a female’s genital area. Brignoli [19] accidentally found an easy technique to assess female genitalia (i.e., spermathecae) when studying booklungs, by dissecting the genital area. Since then, studies on the morphology of schizomids have included only illustrations of the male flagellum and the spermathecae when the females were known, focusing on species identification. This practice last until the 1990’s, when new technologies allowed innovations. Cokendolpher & Reddell [20] published more detailed descriptions of the spermathecae under the light microscope, showing the microtubes of glands attached to the lobes, but it was Santos & Pinto-da-Rocha [21] who first introduced Scanning Electron Microscopy (SEM) into the studies of Neotropical short-tailed whipscorpions. They assessed the ultrastructure of the chelicera, parts of the pedipalp, legs (trichobothria, tarsal claws, cuticular organs), and the male and female flagella. After that, however, most studies continued with the flagellum-spermathecae-only approach, but including newer technologies, such as digital photographs. SEM images have been used since only in a series of studies (chelicera: [22–26]; chelicera and male flagellum: [27, 28]; female flagellum: [24]). Also, Giupponi *et al*. [29] and Villarreal *et al*. [30] published SEM illustrations of the chelicera, pedipalp, male and female flagella and some leg details. Santos *et al*. [31] and Pinto-da-Rocha *et al*. [32], besides presenting SEM images of the flagellum of both sexes, illustrated spermathecae and cuticular structures with SEM. The pygidial gland is known to be present in schizomids since, at least, the beginning of the XX century (e.g. [18]), but to our knowledge have not been assessed in modern morphological works on the order. Regarding the male genitalia, the only work to our knowledge is that by Modder [33], who used only bidimensional cuts to assess internal structures, which resulted in limited understanding and poor comprehension of sclerites of the genital chamber.

*Surazomus algodoal* Ruiz & Valente, 2017 was recently described as the first Neotropical schizomid present in a dry forest, based solely on the male holotype collected from an island near the north coast of Brazil [34]. Since we gathered several new specimens, including previously unknown females and juveniles, and all were perfectly preserved for the use in SEM, we took the chance to improve knowledge on morphology of this species. Hence, our aim in this work is to present further contributions at an ultrastructure level for the setae and cuticle modifications of the chelicera, pedipalp, legs, opisthosoma and pygidial flagellum of both sexes of *S*. *algodoal*. We give illustrations and comments on the pygidial glands and their openings in this species. Moreover, we present here details of the spermathecae and the sexual dimorphism in *S*. *algodoal*. We also use a detailed terminology for the description of the sclerites of the male genital chamber of *S*. *algodoal*, which had never been achieved in schizomids. Such level of detailed description is rare in short-tailed whipscorpions and may help shed light on several biological and phylogenetic issues within Arachnida and arthropods.

## Material and methods

For this work, the male holotype of *S*. *algodoal* was re-examined. Three additional males, two females and five juveniles were collected from the type locality, as follows. Large amounts of litter from a Restinga forest on Maiandeua/Algodoal Island (0.580361°S, 47.582594°W), Maracanã, Pará, Brazil, were put in large plastic bags and taken to an improvised laboratory on the island, where leaves and twigs were searched for the presence of schizomids. Specimens were photographed still alive, collected with a fine brush and preserved in small vials with clear ethanol at 70–80%. New (non-type) specimens are deposited in Universidade Federal do Pará (UFPA). The collecting permit was issued by the Instituto de Desenvolvimento Florestal e da Biodiversidade do Estado do Pará (IDEFLOR-Bio, processes 217054/2018 and 005/2019), and ethical approval for this study was waived by the Comissão de Ética no Uso de Animais of the Universidade Federal do Pará (CEUA-UFPA), because the study does not include any vertebrate taxon.

The material was primarily studied in ethanol in a Petri dish under a Leica M205A microscope. The male holotype was used for the description of palpal setae. The palp was dissected and immersed in clove oil. One male (UFPA-SCH-001) was used for the study of genital sclerites under light microscopy. The genitalia were dissected and put directly in Hoyer’s medium in separate slides and covered with a coverslip; after letting slides dry in a stove for three days, coverslips were sealed with transparent nail polish; this specimen was also used for comparison of palpal setae with the holotype. The genitalia of a second male (UFPA-SCH-002) were put in pancreatin to remove soft tissues and used in SEM images and, after that, rehydrated and lips of the gonotreme were separate and immersed in clove oil for the study of genital sclerites under light microscope. All three males (UFPA-SCH-001-003) were used for variation of flagellar microsetae under light microscopy. One female (UFPA-SCH-004) was used for measurements and for the study of the pedipalp and spermathecae, dissected and put directly in Hoyer’s medium, as for the male. A second female (UFPA-SCH-005) was used for SEM images (spermathecae were mounted for SEM, but did not render good illustrations); also, the opisthosoma was macerated with KOH to assess the pygidial glands under light miscroscopy, following Hansen & Sørensen [18]. Two juveniles (UFPA-SCH-006, dorsal, and UFPA-SCH-007, lateral) were used in SEM images.

Live animals were photographed with an Olympus IM006 camera, using a M. Zuiko 60 mm macro lens. Drawings were prepared with a Leica M205A microscope (male flagella and ventral male pedipalp) and a Leica DM 1000 microscope (lateral male pedipalp, male and female genitalia, female flagellum), both with a camera lucida. Multifocus colour photographs were taken with a Leica DFC420 digital camera attached to a Leica M205A microscope (pygidial gland), and an AmScope MU1403-CK camera attached to a Leica DM 1000 microscope (male and female genitalia), all stacked with Helicon Focus 7.6.6 Pro software. Scanning electron micrographs (SEM) were taken with a Zeiss Leo 1450 VP microscope in the Laboratório Institucional de Microscopia Eletrônica de Varredura of the Museu Paraense Emílio Goeldi (female chelicera and legs, male and female pedipalps and flagella, which were dried with critical point before SEM), and a Tescan Vega 3 microscope at Laboratório de Biologia Estrutural of the Instituto de Ciências Biológicas, Universidade Federal do Pará (male genitalia and juveniles). Measurements, expressed in millimeters, were taken with a Leica M205A microscope, using Leica Application Suite.

Terminology follows Villarreal *et al*. [27, 30] for opisthosomal setation, and Lawrence [35], modified by Villarreal *et al*. [30], for cheliceral setation. Pedipalp setation follows Monjaraz-Ruedas & Francke [23, 24] and Ruiz & Valente [3], including the new terminology for setae of the pedipalpal trochanter: medial (TRm), ventral (TRv), subectal (TRs) and ectal (TRe) lines. Monjaraz-Ruedas & Francke’s [24] setae of the Tibial ectal row (Ter), Tibial medial row (Tmr), and Tibial internal row (Tir) are herein referred individually (Te, Tm, Ti). Monjaraz-Ruedas & Francke’s [24] Tibial mesal (Tm1 and 2) setae are renamed herein as Tibial ventral (Tv). We also use the new terminology for plumose setae of the pedipalpal tarsus: medial row (TAm) and internal row (TAi). The terminology of flagellar setation follows Monjaraz-Ruedas *et al*. [36]. Female genitalia are described using our own terminology, modified from Brignoli’s [19]. Male genitalia are described using Modder’s [33] and Giupponi and Kury’s [37] terminology, when applicable. Most terms are newly proposed: Gonosternite; Median apodeme (MA); Furcula (Fu; with Dorsal and Ventral arms–DAFu / VAFu; and Lateral bar–LBFu); Lateral flap (LF); Papillate flap (PF); Median field (MF); Median septum (MS); Pterapophysis (Pt); Hook of fistula (H); Bridge (Br); Fold of gonopod (FGp). We also propose names for the muscles of male genitalia: Anterior (AM); Posterior (PM); Anterior Transverse (ATM); Posterior Transverse (PTM); Oblique (OM).

Definition of trichobothria follows that by Dahl [38]; Slit organ and lyriform organ by Bertkau [39] and Gaubert [40], respectively. Slit-like and rosette-shaped glandular openings by Seiter *et al*. [41]. We also propose the following names for different structures, as follows: rimmed pore; vaporizer sclerite (VS).

Other abbreviations used throughout: Coxa (Cx); Trochanter (TR); Femur (F); Patella (P); Tibia (T); Tarsus (TA); Gonotreme (Gt); Dorsal lip of gonotreme (DLGt); Ventral lip of gonotreme (VLGt); Fistula (Fi); *Lobus lateralis primus* (LoL1); *Lobus lateralis secundus* (LoL2); *Lamina medialis* (LaM).

## Results

### Description of the female of *S*. *algodoal*

#### Description

**Fig 1:**
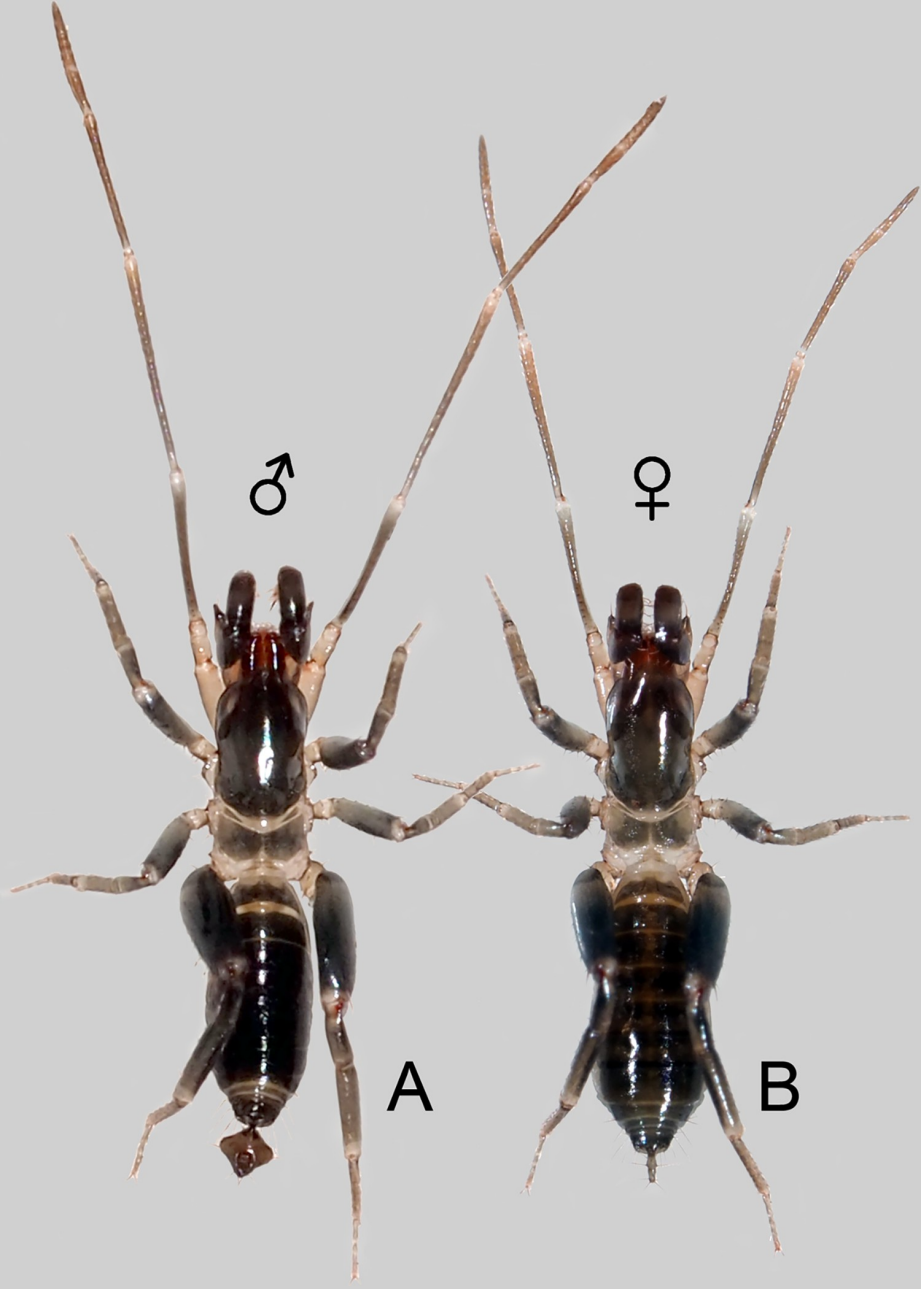
Live specimens of *Surazomus algodoal*. Dorsal view: (A) male, (B) female.

**Fig 2:**
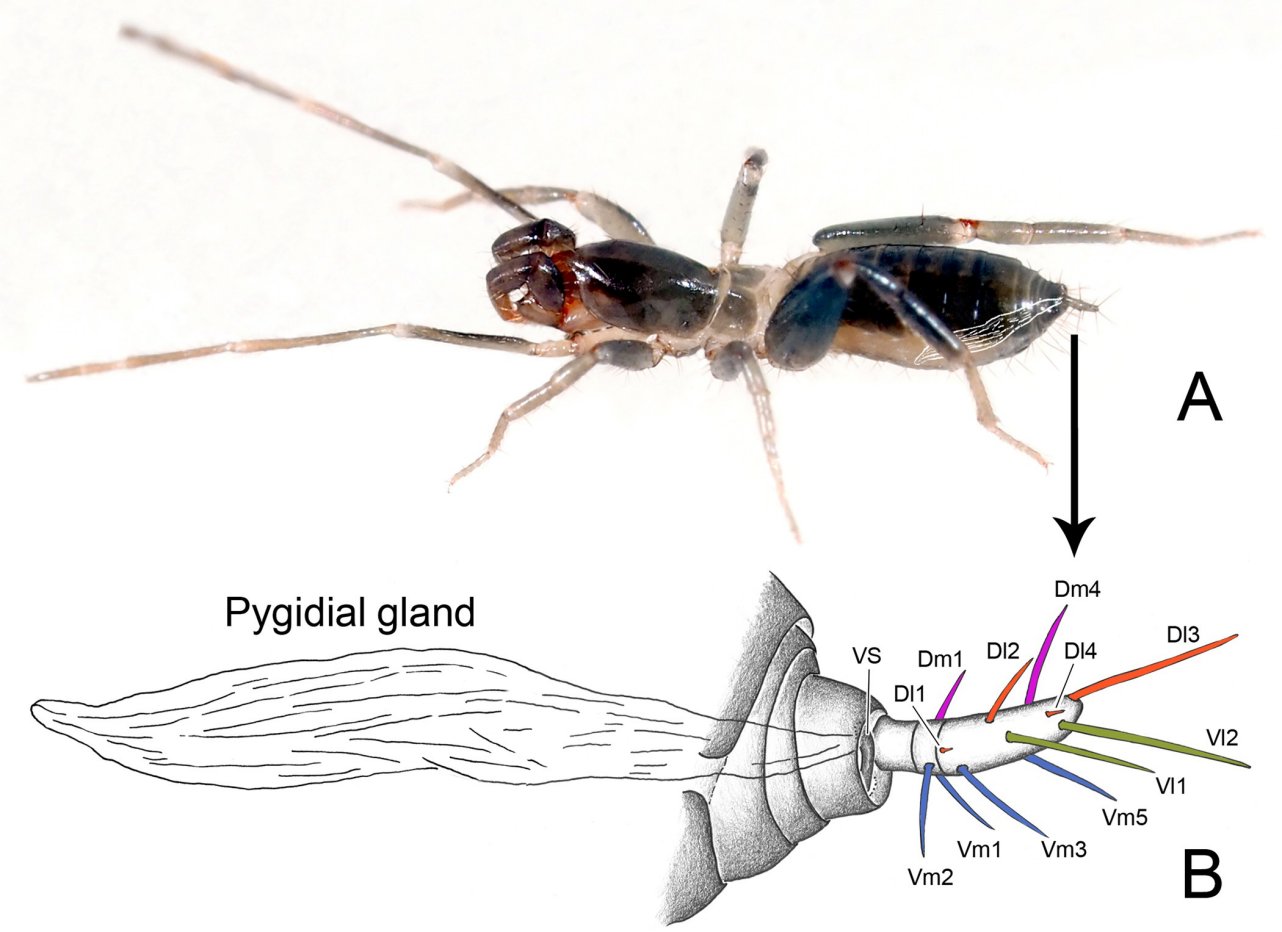
*Surazomus algodoal*. (A) live female, lateral view (internal pygidial gland shown), (B) detail of posterior portion of opisthosoma, showing pygidial gland and setae of flagellum. Abbreviation: (VS) vaporizer sclerite. For flagellar setae, see text.

**Fig 3:**
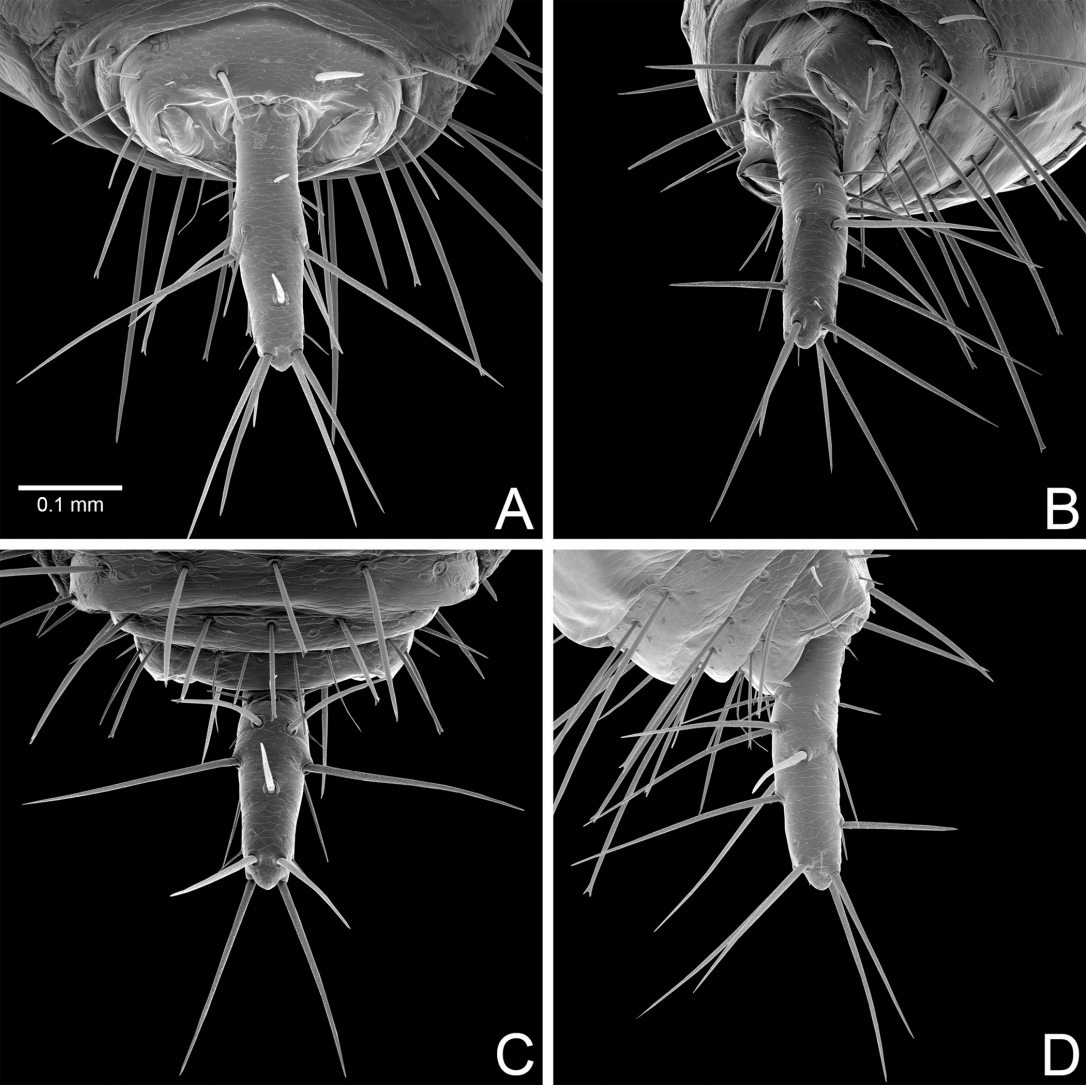
*Surazomus algodoal*, female. (A-D) pygidial flagellum: (A) dorsal view, (B) dorsolateral view, (C) ventral view, (D) lateral view.

**Fig 4:**
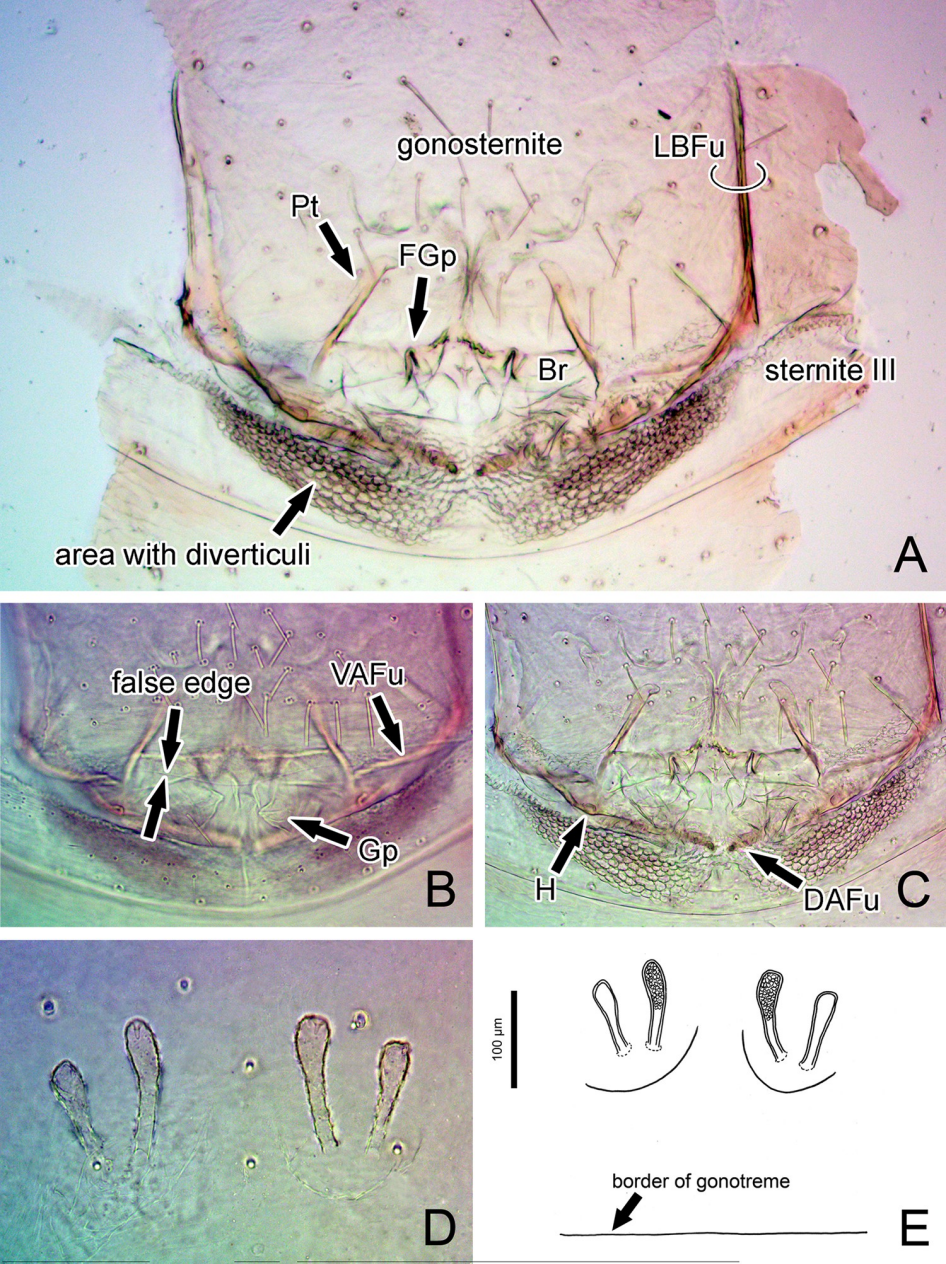
Genitalia of *Surazomus algodoal*. (A-C) male, cleared: (A) ventral view, (B) dorsal view, (C) ventral view, (D-E) female, ventral view. Abbreviations: (Br) bridge, (DAFu) dorsal arm of furcula, (Gp) gonopod, (FGp) fold of gonopod, (H) hook of fistula, (LBFu) lateral bar of furcula, (Pt) pterapophysis, (VAFu) ventral arm of furcula.

Total length: 3.2, not including the flagellum. Prosoma 1.35 long; propeltidium: 1.05 long. The female is similar to the male in general appearance (Fig 1; see Ruiz & Valente [34]), except for: Pedipalps with less evident apical process on trochanter and no apophyses on femur and patella (Figs 9B, 10C, 10E). Leg I shorter (about 3.8x the prosoma in males and 3.0x the prosoma in females; Fig 1A, 1B). Opisthosoma 1.2x larger than in male; with no posterodorsal process on tergite XII (Figs 2B, 3; for comparison with male, see Fig 16); posterior border of sternite II straight (Fig 4E; lobed in males; see Ruiz & Valente [34]: fig 4). Flagellum (Figs 2 and 3) 0.38 long, 0.07 wide (n = 1), with two annuli (between flagellomeres I-II and II-III); flagellomeres III+V-VI are fused (Fig 2B); flagellomere I: no setae; flagellomere II: Dm1 aligned with Vm2 (pair) and Vm1; flagellomeres III+V-VI: microseta Dl1 (pair) aligned with Vm3 (pair), Dl2 (pair) aligned with Vl1 (pair), Dm4 and Vm5 present, Dl3 (pair) aligned with Vl2 (pair), microseta Dl4 (pair) present. Spermathecae (Fig 4D, 4E): two pairs of spermathecae on low protuberance, each consisting of a posterior straight tube and an anterior rounded bulb, less than 1.5 the diameter of the tube, all covered with numerous inconspicuous “duct openings”. Median spermathecae slightly longer than lateral ones, with a single papilla on the distal part of inner surface. Chitinized arches absent.

#### Diagnosis

The female of *S*. *algodoal* is similar to those of *S*. *antonioi* Armas & Víquez, 2014, *S*. *boliviensis* Cokendolpher & Reddell, 2000, *S*. *chiapasensis* Monjaraz-Ruedas, Prendini & Francke, 2020, *S*. *cumbalensis* Kraus, 1957, and *S*. *palipatellatus* Rowland & Reddell, 1979 for having a set of four spermathecae with the bulb about the same girth of the tube, with no final constriction, but can be distinguished from those of *S*. *antonioi*, *S*. *boliviensis*, and *S*. *cumbalensis* for having the median spermathecae longer than the lateral ones, from *S*. *chiapasensis* for not having a group of duct openings between the spermathecae, and from *S*. *palipatellatus* for not having a sinuous tube in the lateral spermathecae (Fig 4D, 4E; see Armas & Víquez [42]: figs 3B-C; Cokendolpher & Reddell [43]: fig 16; Monjaraz-Ruedas *et al*. [44]: fig 8A; Villarreal *et al*. [30]: fig 8D; Rowland & Reddell [45]: fig 66).

### Description of male genitalia of *S*. *algodoal*

#### Description

**Fig 5:**
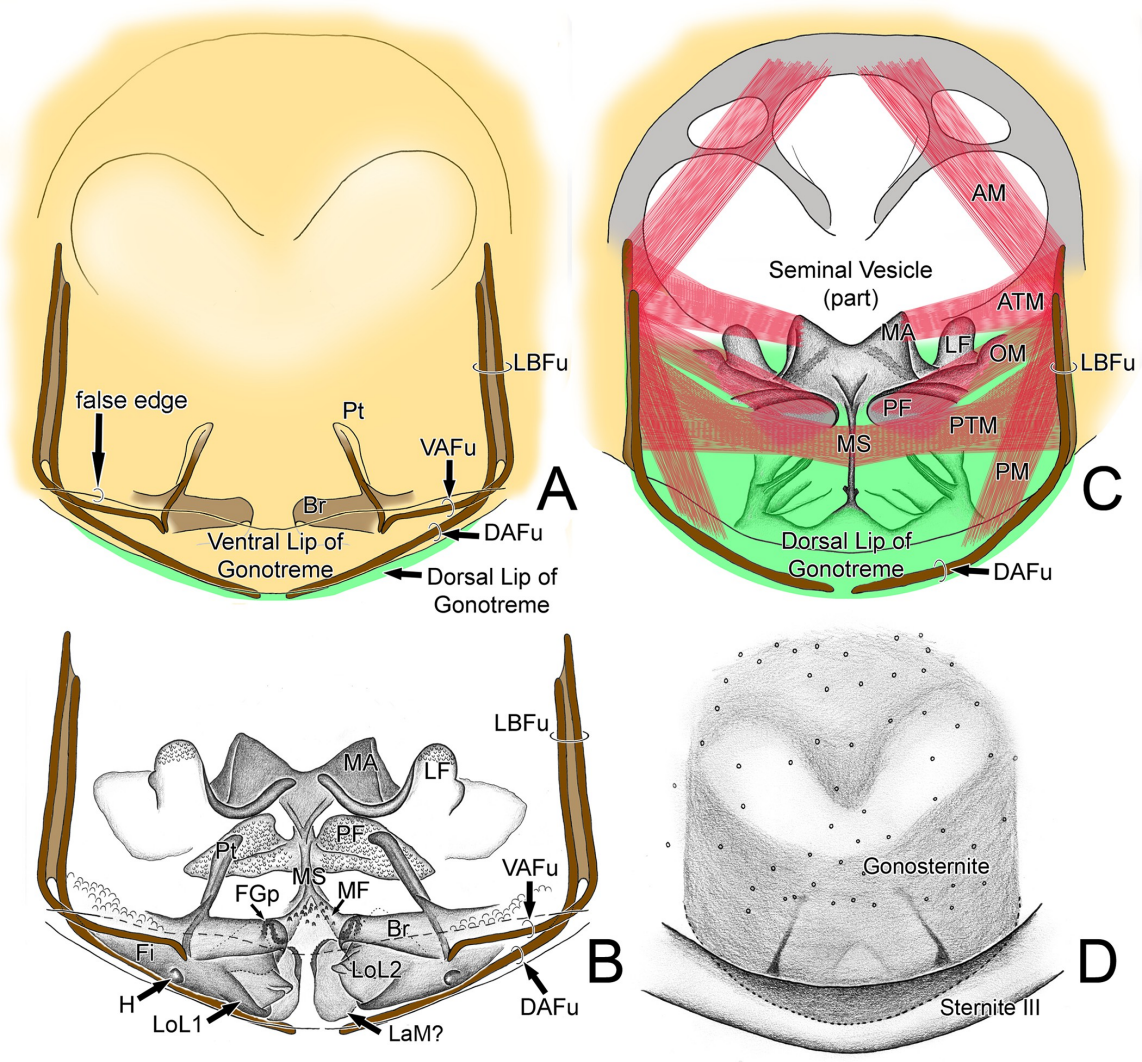
Male genitalia of *Surazomus algodoal*. (A-B and D) ventral view, (C) dorsal view. Abbreviations: (AM) anterior muscle, (ATM) anterior transverse muscle, (Br) bridge, (DAFu) dorsal arm of furcula, (FGp) fold of gonopod, (Fi) fistula, (H) hook of fistula, (LaM) *lamina medialis*, (LBFu) lateral bar of furcula, (LF) lateral flap, (LoL1) *lobus lateralis primus*, (LoL2) *lobus lateralis secundus*, (MA) median apodeme, (MF) median field, (MS) median septum, (OM) oblique muscle, (PF) papillate flap, (PM) posterior muscle, (Pt) pterapophysis, (PTM) posterior transverse muscle, (VAFu) ventral arm of furcula.

**Fig 6:**
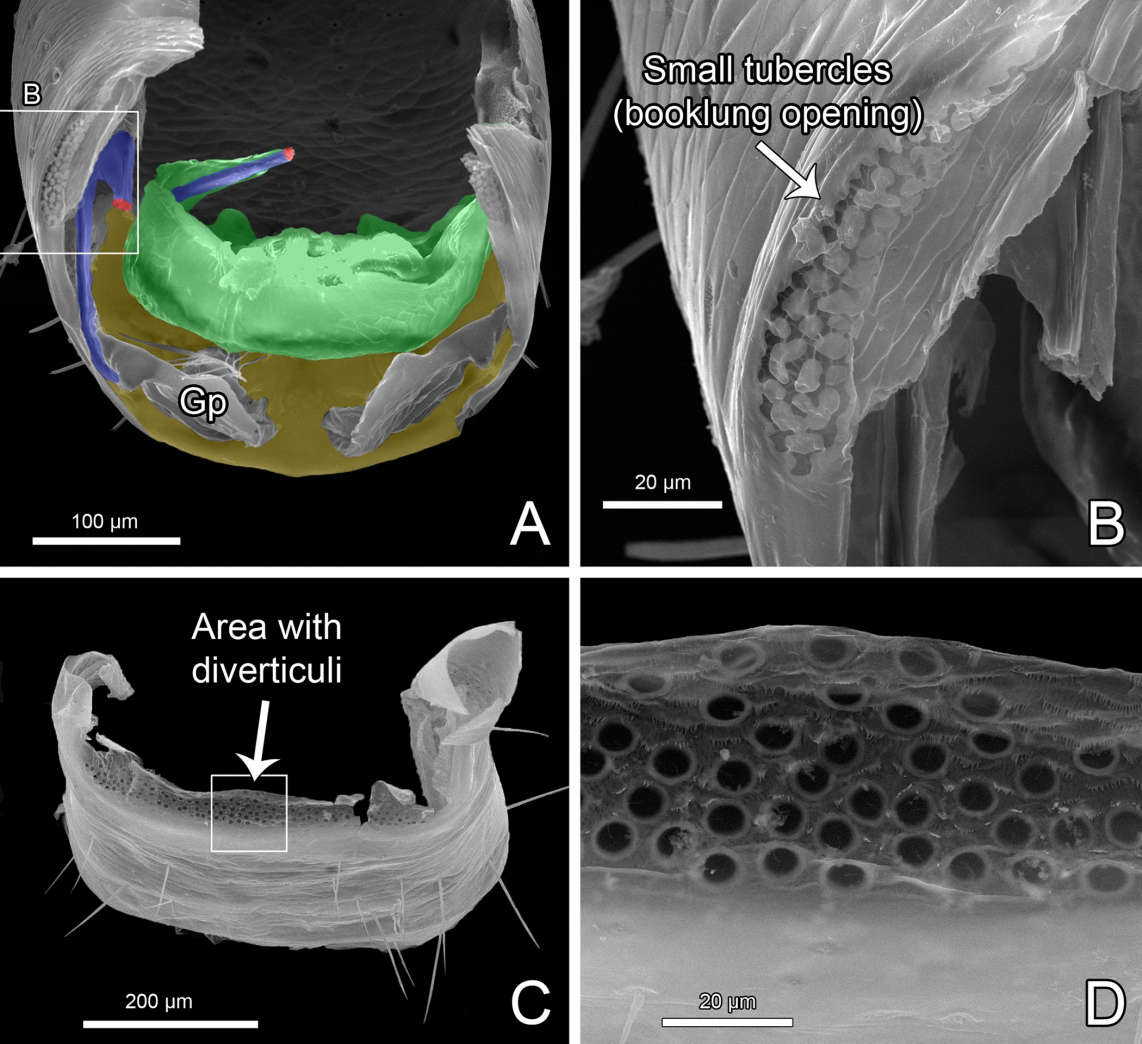
Male genitalia of *Surazomus algodoal*. (A) gonosternite, dorsal view (ventral lip of gonotreme in yellowish brown; dorsal lip of gonotreme in green; furcula in blue; two red markings show fracture of furcula holding dorsal lip), (B) detail of previous showing tubercles along booklung opening, (C) sternite III, antero-ventral view, (D) detail of previous showing diverticuli on anterior border of sternite. Abbreviation: (Gp) gonopod.

**Fig 7:**
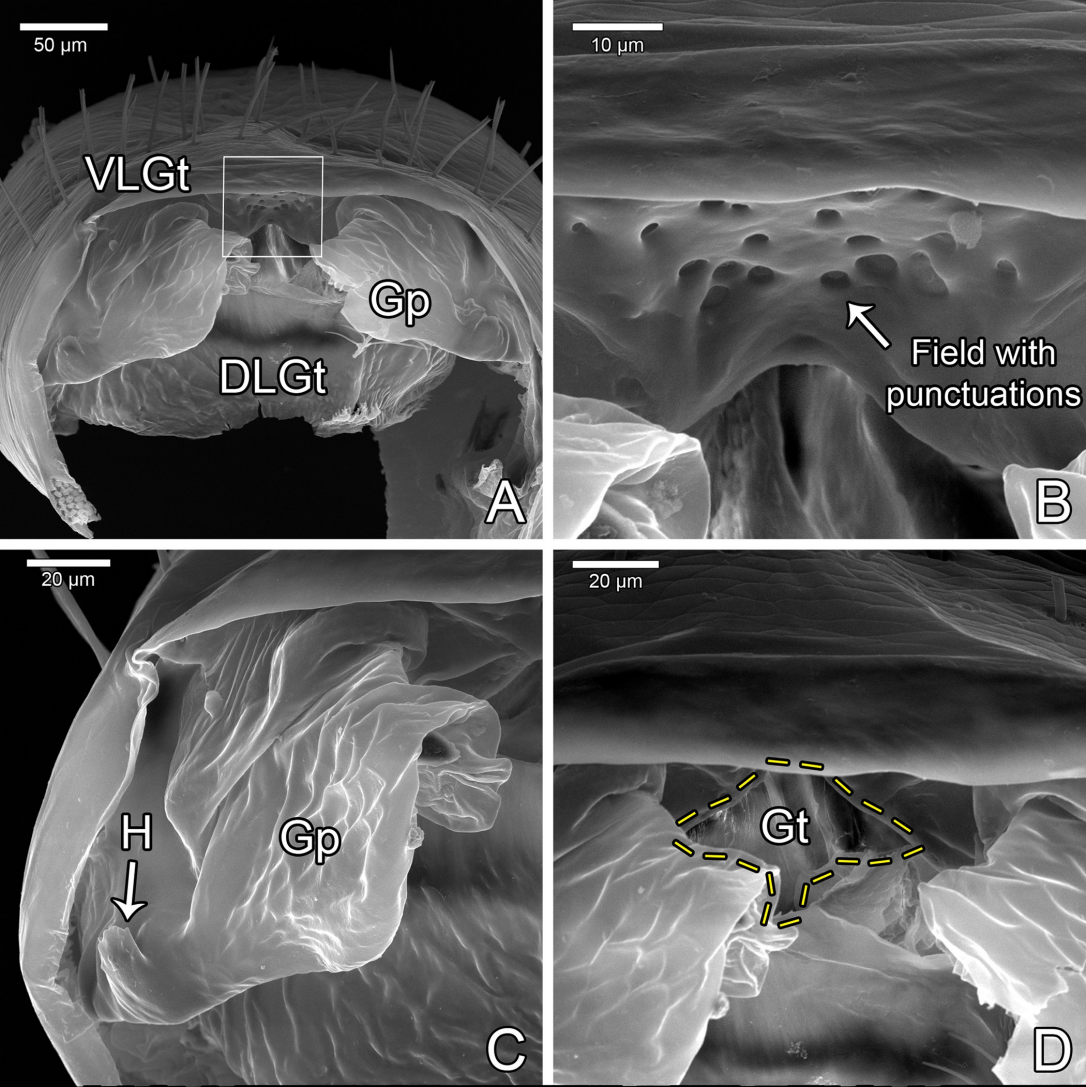
Male genitalia of *Surazomus algodoal*. (A) gonosternite, posterior view, (B) detail of previous showing punctuations on median septum, (C) detail of previous showing gonopod and fistula, with hook, (D) detail of previous showing base of gonopods beneath gonotreme. Abbreviations: (DLGt) dorsal lip of gonotreme, (Gp) gonopod, (Gt) gonotreme, (H) hook of fistula, (VLGt) ventral lip of gonotreme.

Gonosternite (opisthosomal sternite II) large, occupying over 1/3 of opisthosomal length, entirely covered with short setae (Figs 4A, 5D, 7A; see also Ruiz & Valente [34]: fig 4). Anterior half of gonosternite with a pair of whitish areas (part of seminal vesicle, see below) and posterior third with a pair of oblique dark stripes (pterapophyses (Pt), see below) seen through the cuticle (Figs 4A–4C, 5D, see also Ruiz & Valente [34]: fig 4). Border along booklung openings covered with small tubercles (Fig 6A, 6B). Median portion of posterior border of gonosternite hidden under sternite III (Figs 4A, 5D), forming a false edge (Figs 4B, 5A-5C). Opisthosomal sternite III with a pair of areas covered with small diverticuli (Figs 4A, 4C, 6C, 6D). **Genital structures** (Table 4): anterior half of gonosternite with partly preserved seminal vesicle (ventral), consisting of three roundish pouches (one median and a pair of lateral ones, Fig 5A, 5C). Remaining portions of seminal vesicle (dorsal) not preserved in specimen analyzed. Posterior half of gonosternite bearing the genital chamber, a dorsoventrally compressed space delimited by the ventral (VLGt) and the dorsal (DLGt) lips of gonotreme (Gt) (Figs 5A–5C, 6A, 7A, 7D). The genital chamber is reinforced by a complex sclerite herein named as the furcula (Fig 6A), which has a pair of flattened longitudinal lateral bars (LBFu) acting as an apodeme for several muscles (Figs 4A, 5A-5C). These bars bifurcate posteriorly and form the ventral and the dorsal arms (VAFu and DAFu, respectively), which reinforce the respective lips of gonotreme (VLGt and DLGt) (Figs 4A, 5A-5C, 6A, 7A). **Ventral lip of gonotreme** (Figs 5A, 6A, 7A): the ventral arms of furcula (VAFu) run transversely near the surface of the gonosternite and meet submedially the base of the pterapophyses (Pt), a pair of oblique flattened, slightly clavate sclerites attached to cuticle of the gonosternite (Figs 4A–4C, 5A, 5B). Pterapophyses (Pt) extend medially and ventrally as the bridges (Br), a thin pair of ventral sclerites that anchor the gonopods (Figs 4A, 4B, 5A, 5B). The bridges are joined by a median field (MF) with a short longitudinal median septum (MS) and small tubercles (Fig 5B), externally seen on the surface as punctuations (Fig 7A, 7B). From the median field, a pair of membranes extend loosely, form a pair of sclerotized folds of the gonopods (FGp) and then turn into the base of the gonopods (Gp = Fi+LoL1+LoL2+LAM), which hang from the ventral lip and are kept inside the chamber along the border of the gonosternite (Figs 4A, 4B, 5B, 6A, 7A, 7C). The external base of the gonopods sclerotized and identified as the fistula (Fi) (Fig 5B). Fistula with a hook (H) pointing posteriorly/medially (Figs 4C, 5B, 7C). Distal portion of gonopods membranous, with three lobes (Fig 5B): ventrally with *lobus lateralis primus* (LoL1) followed by *lobus lateralis secundus* (LoL2), covered dorsally by a possible *lamina medialis* (LaM). LoL2 very developed and wrapped, with several folds (Fig 5B). Anterior border of LaM serrated (Fig 5B). **Dorsal lip of gonotreme** (Figs 5A, 5C, 6A, 7A): dorsal arm of furcula (DAFu) projecting medially, forming the border of the dorsal lip (Fig 5A–5C). Anteriorly with a pair of median apodemes (MA) joined with a pair of bilobed lateral flaps (LF) by a concave surface, formed by an extension of the border of the lateral flaps (Fig 5B, 5C). A pair of papillate flaps (PF) right posterior of the lateral flaps (Fig 5B, 5C), with papillae pointing towards the interior of the genital chamber. Median septum (MS) extending longitudinally from the posterior border to the center of the genitalia, slightly passing the median apodemes (MA) (Fig 5B, 5C). MA and LF covered ventrally with short spicules (Fig 5B), and posterior border of dorsal lip internally covered with large spicules. **Muscles** (Fig 5C): Anterior Muscles (AM) connected from the area anterior to the seminal vesicle to LBFu. Posterior Muscles (PM) from LBFu to posterior border (gonopods?). Anterior Transverse Muscles (ATM) from the pair of MA to the LBFu. Posterior Transverse Muscles (PTM) from the LBFu to the median septum. Oblique Muscles (OM) from the papillate flaps to LBFu.

### Revision of setae of *S*. *algodoal*

The examination of ultrastructure of setae on the body of *S*. *algodoal* revealed six patterns of microtrichium ornament along the seta shaft (Table 1). We understand that these patterns may vary among different setae of a single seta group and along a single seta, but we suggest that it is worthy coding these patterns when data are available.

**Table 1:** Classification and distribution of setae according of microtrichium patterns along seta shaft in *S*. *algodoal*.

Seta name	Description	Distribution
Smooth seta (Fig 13C)	No microtrichia	Only on tip of leg I
Barbed seta (Fig 8F)	Covered with short microtrichia on the entire circumference of the seta, including flagellar type A of Villarreal *et al*. [30]	Over entire body (chelicera G6 type)
Pectinate seta (Fig 8E)	Bearing long microtrichia along one side of the seta only	Only on chelicera (G4, middle portion of G5B, G7 types)
Bipectinate seta (Fig 8B)	Bearing long microtrichia primarily on opposite sides of the seta	Only on chelicera (G2 type)
Brush (Fig 10G)	With long microtrichia covering one entire face of the seta, distributed in many lines.	Chelicera (G1 type), prolateral face of palpal patella, tibia and tarsus
Plumose seta (Fig 8D)	With long microtrichia covering the entire circumference of the seta, distributed in many lines, including flagellar type B of Villarreal *et al*. [30]	Chelicera (G3, G5A, distal portion of G5B types)

#### Chelicera

**Fig 8:**
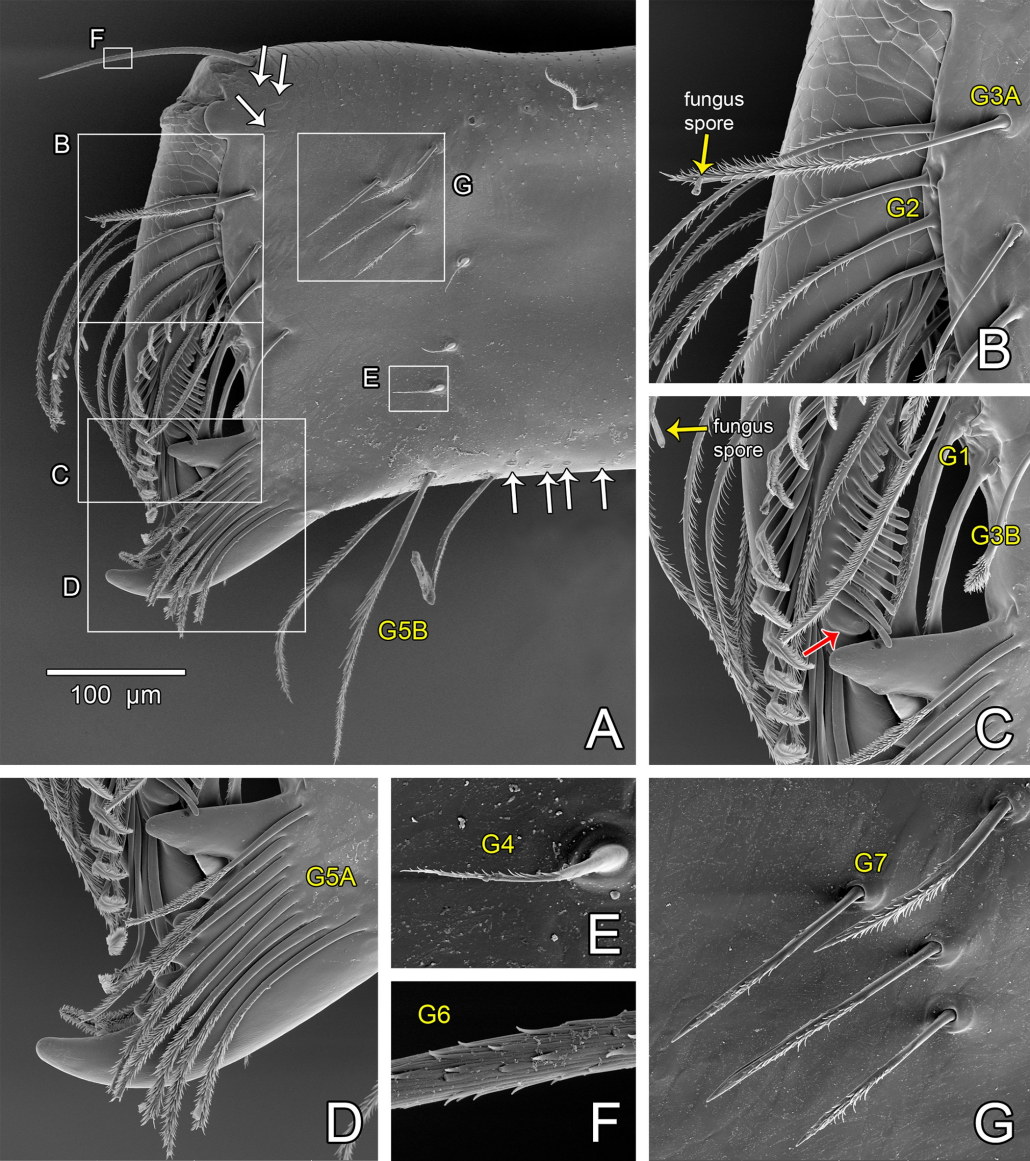
Right chelicera of the female of *Surazomus algodoal*, showing seta types. (A) prolateral view (white boxes shown in more details in following illustrations; white arrows show slit sensilla; yellow names refer to seta type), (B) detail showing bases of setae G2 and G3A, (C) detail showing hyaline teeth and guard tooth (red arrow), bases of setae G1 and G3B, (D) detail of fixed finger with setae G5A, (E) detail of one G4 seta, (F) detail of a segment of G6 seta, (G) detail showing G7 setae. All prolateral view.

*Surazomus algodoal* (Fig 1A, 1B) had setae of the chelicera (male holotype) described in detail concerning types and numbers (Ruiz & Valente [34]: fig 8). However, those details were limited to observation under light microscopy (400x), with the structure immersed in clove oil. We assume that differences found herein in the morphology of the setae of the chelicera in SEM (Fig 8) (Table 2) may be due to that limitation.

**Table 2:** Update in knowledge on setae present on the chelicera of *S*. *algodoal*.

Chelicera seta type	Light microscope		SEM
Villarreal *et al*. [30]	Ruiz & Valente [34]	(new)	(new)
Group 1 (G1)	3 spatulate setae, peduncle smooth	hollow (in clove oil)	3 (wide) brush, stalk smooth (Fig 8A, 8C)
Group 2 (G2)	5 subequal feathered setae, as long as movable finger	hollow (in clove oil)	5 bipectinate (Fig 8A–8C), stalk smooth, same length
Group 3 (G3)	4 setae	-	4 plumose, stalk smooth (Fig 8A–8C) *, hollow
Group 4 (G4)	3 smooth, short and thick setae with thin apex	-	3 pectinate, same shape (Fig 8A, 8E)
Group 5 (G5A)	7 similar-sized feathered setae, as long as distal tooth	-	8 plumose, stalk smooth, same length (Fig 8A, 8D), hollow
Group 5 (G5B)	6 + 2, long, smooth and straight	hollow (in clove oil)	3 pectinate middle portion, distally plumose, stalk smooth (Fig 8A)
Group 6 (G6)	1, smooth, longer than half the length of the movable finger	-	1, barbed, about half the length of the movable finger (Fig 8A, 8F)
Group 7 (G7)	6, equal in size	-	7 pectinate, stalk smooth, same size (Fig 8A, 8G)
Formula	3-5-4-3-(7+8)-1-6		3-5-4-3-(8+3)-1-7

*Note. Based on the SEM illustrations, herein we distinguish two types of G3 setae: type G3 A (Fig 8B), longer and with the plumose portion about as long as the stalk; and type G3 B (Fig 8C), shorter, with the plumose portion shorter than the stalk.

Other than the setae, our SEM illustrations allow us to observe in details: 1) the guard tooth of the movable finger (red arrow in Fig 8C); 2) the serrula of the movable finger with 14 hyaline teeth (Fig 8C); 3) the row with 18 stalked plumose setae of the movable finger (Fig 8C); 4) the basal tooth of the fixed finger not bifid (bifid in the male holotype), followed by five small teeth and a larger, recurved tooth, with an acute apex (Fig 8D).

#### Pedipalp

**Fig 9:**
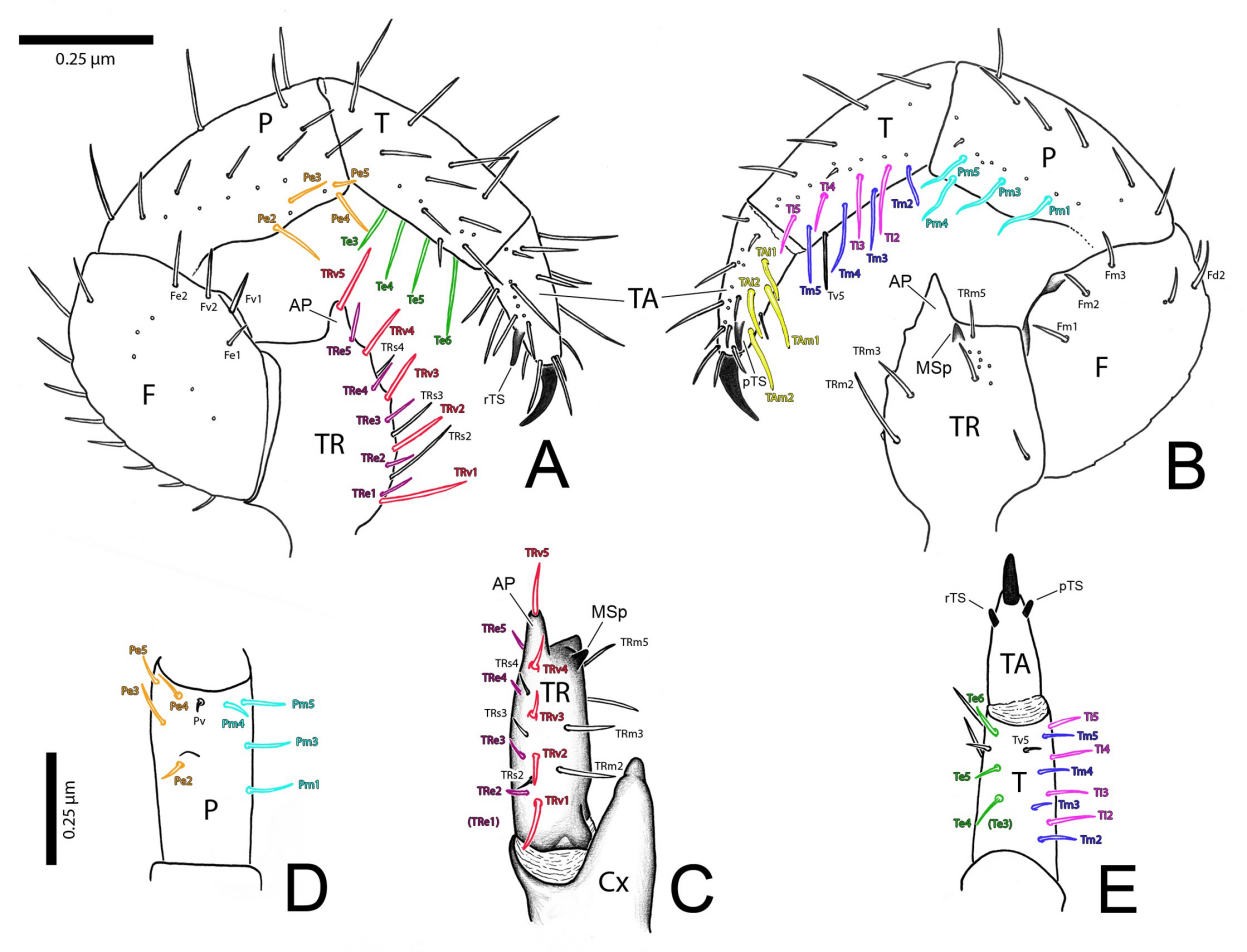
Right male pedipalp of *Surazomus algodoal*. (A-B) holotype: (A) retrolateral view, (B) prolateral view, (C-E) male UFPA-SCH-001: (C) trochanter, ventral view, (D) patella, ventral view, (E) tibia, ventral view. Different colors show seta groups. Abbreviations: (AP) apical process, (Cx) coxa, (F) femur, (MSp) mesal spur, (P) patella, (pTS) prolateral tarsal spur, (rTS) retrolateral tarsal spur, (T) tibia, (TA) tarsus, (TR) trochanter. For seta names, see text. Seta names in parentheses show setae present in the holotype, but absent in male UFPA-SCH-001.

**Fig 10:**
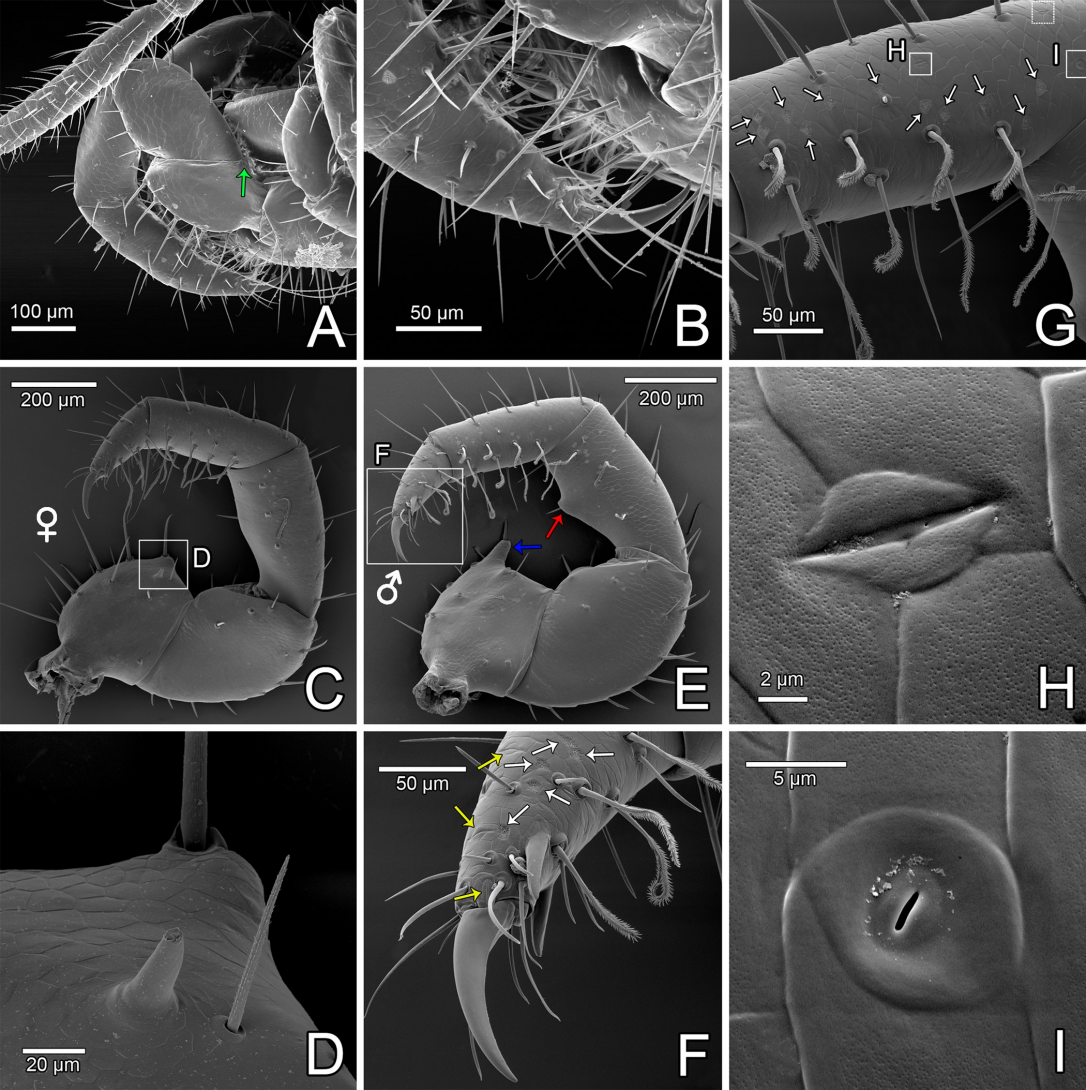
Pedipalp of *Surazomus algodoal*. (A) juvenile, left pedipalp, retrolateral view (green arrow shows lyriform organ), (B) detail of previous illustration showing tarsal spurs, (C) female, right pedipalp (detached), prolateral view, (D) detail showing mesal spur, (E) male, right pedipalp (detached), prolateral view (male dimorphic apophyses shown by blue arrow on trochanter and by red arrow on patella), (F) detail of tarsus, showing cuticular organs (white arrows show rosette-shaped glandular openings; yellow arrows show slit sensilla), (G) tibia, prolateral view (male), showing cuticular organs (white arrows show rosette-shaped glandular openings), (H) slit sensillum, (I), slit-like glandular opening.

The pedipalp of the male holotype of *S*. *algodoal* has been described ([34]: fig 7), but its setae have not. These are described herein (Fig 9) and compared with SEM illustrations of another male, female and juvenile (Fig 10).

Herein we also propose a new terminology to describe setae of the pedipalpal trochanter and plumose setae of the pedipalpal tarsus (Fig 9A–9C). On the trochanter, setae are named as forming four longitudinal lines, based on a pattern of 5 setae per line: medial (TRm), ventral (TRv), subectal (TRs) and ectal (TRe) lines. The plumose setae of the pedipalpal tarsus are disposed in two longitudinal lines: setae of the medial row (TAm) and the internal row (TAi), to match alignment with the tibial setae. Setae of pedipalp (male holotype: Fig 9A, 9B). Trochanter with TRm2, TRm3 and TRm5; TRv1-TRv5; TRs2-TRs4; TRe1-TRe5 (male UFPA-SCH-001: TRe2-TRe5: Fig 9C). Femur with a dorsal row of nine setae; retrolaterally with Fe1, Fv1, Fv2, Fe2 and one additional, subdistal, subdorsal seta; prolaterally with Fm1-3, and with Fd2 and two additional setae. Patella dorsally with three setae; retrolaterally with Pe2 (on PAP–patellar apophysis) and Pe3-Pe5 distally, and seven other unnamed setae; prolaterally with long and sinuous Pm1, Pm3-Pm5 (as in male UFPA-SCH-001: Fig 9D); additionally with seven unnamed setae. Tibia with Te3-Te6 (male UFPA-SCH-001: Te4-Te6: Fig 9E), Ti2-Ti5, Tm2-Tm5 and Tv5 present; additionally, there are several other dorsal, prolateral and retrolateral unnamed setae on the tibia. Tarsus (Fig 9B) with TAm1-2 and TAi1-2, besides several other unnamed setae.

**Fig 11:**
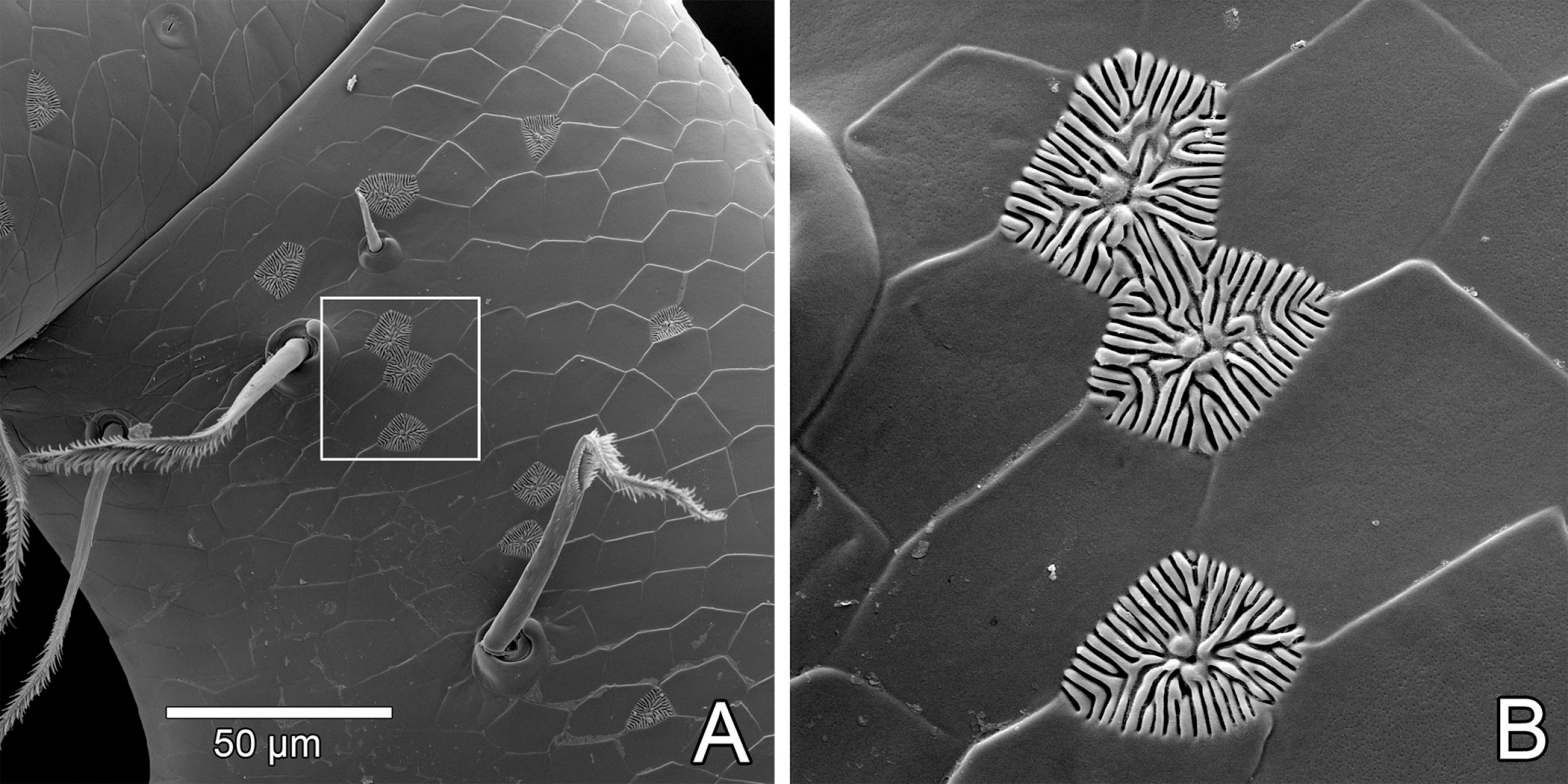
Pedipalp of *Surazomus algodoal*. (A) male, patella of right pedipalp, prolateral view, (B) detail of previous illustration showing rosette-shaped glandular openings.

Setae present along the meso-ventral stripe on palpal patella (Pm), tibia (Ti, Tm and Tv) and tarsus (TAi and TAm) are long, stalked, with the distal half as brushes in both sexes (Figs 10C, 10E–10G, 11A). The remaining palpal setae are barbed (Fig 10A, 10B).

#### Legs

**Fig 12:**
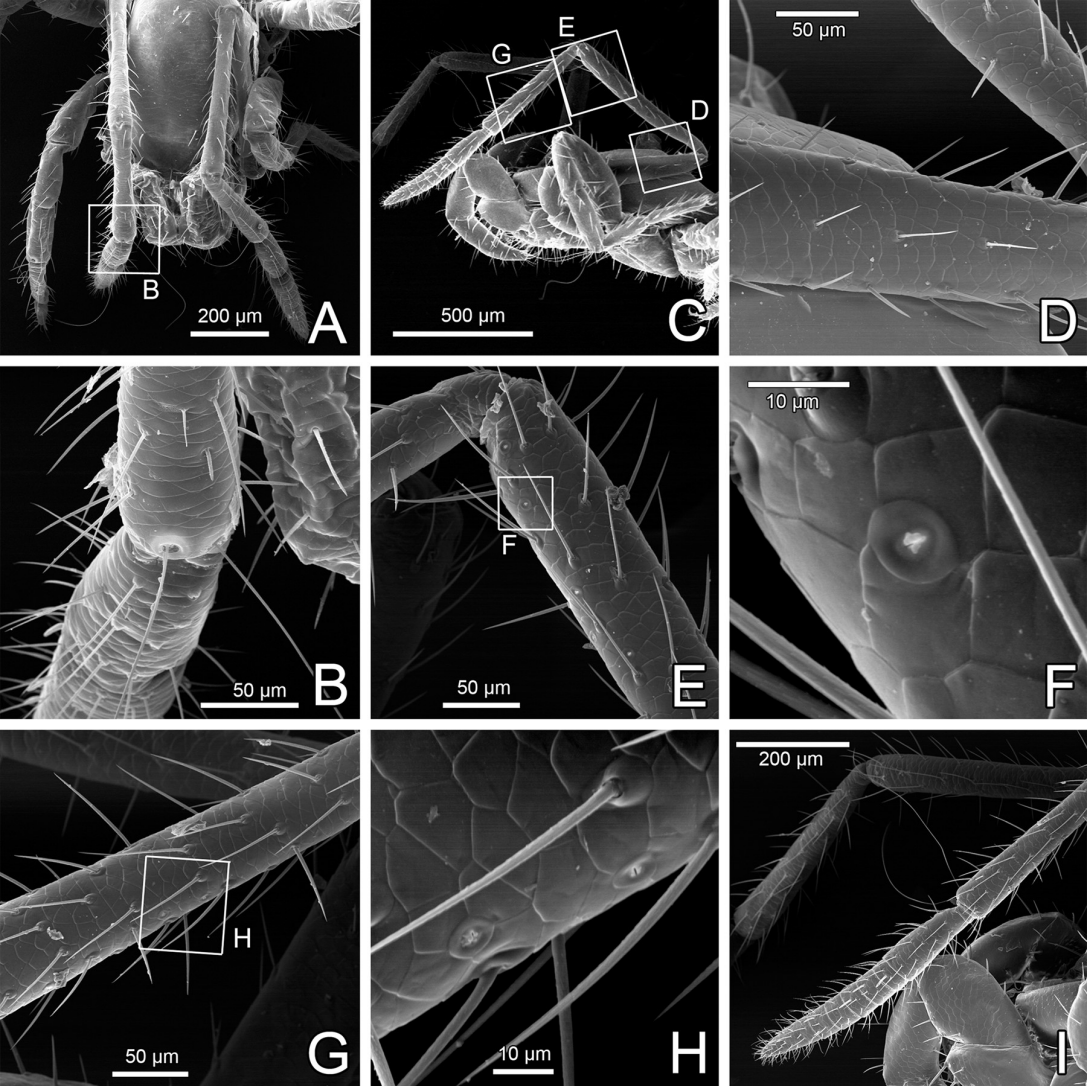
Legs of *Surazomus algodoal*. (A) juvenile, anterior legs, dorsal view, (B) detail of previous illustration showing pair of trichobothria on tibia I, (C) juvenile, anterior legs, retrolateral view, (D) femur of leg I, (E) tip of patella of leg I, (F) detail of previous showing slit-like glandular opening, (G) tibia of leg I, (H) detail of previous showing slit-like glandular openings, (I) tip of leg I.

**Fig 13:**
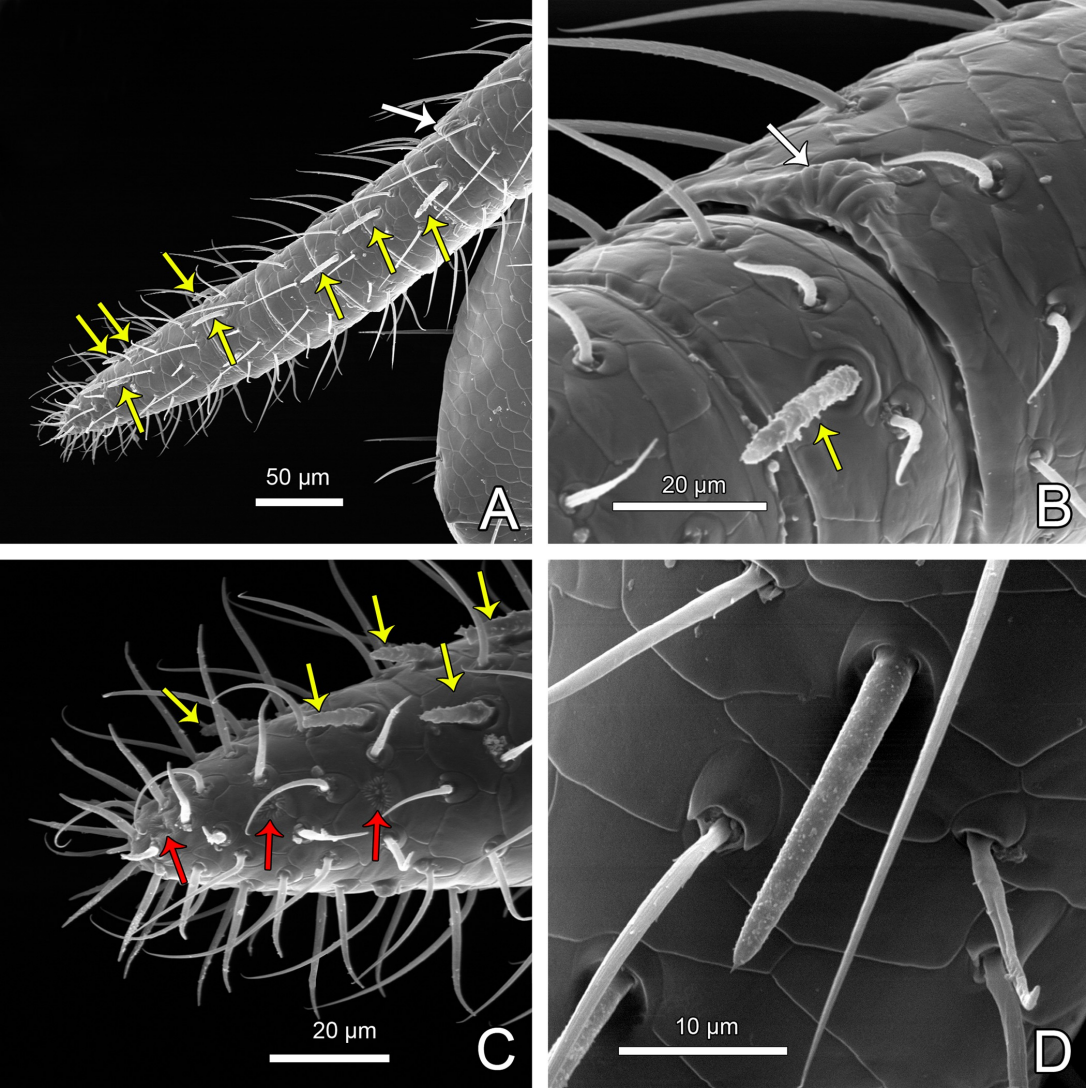
Tip of leg I of *Surazomus algodoal*. (A) tarsomeres, retrolateral view, (B) articulation metatarsus-tarsus of leg I, dorsal view, (C) tip of leg I, (D) detail of club seta. Yellow arrows: club setae; Red arrows: rosette-shaped glandular opening; White arrows: lyriform organ.

**Fig 14:**
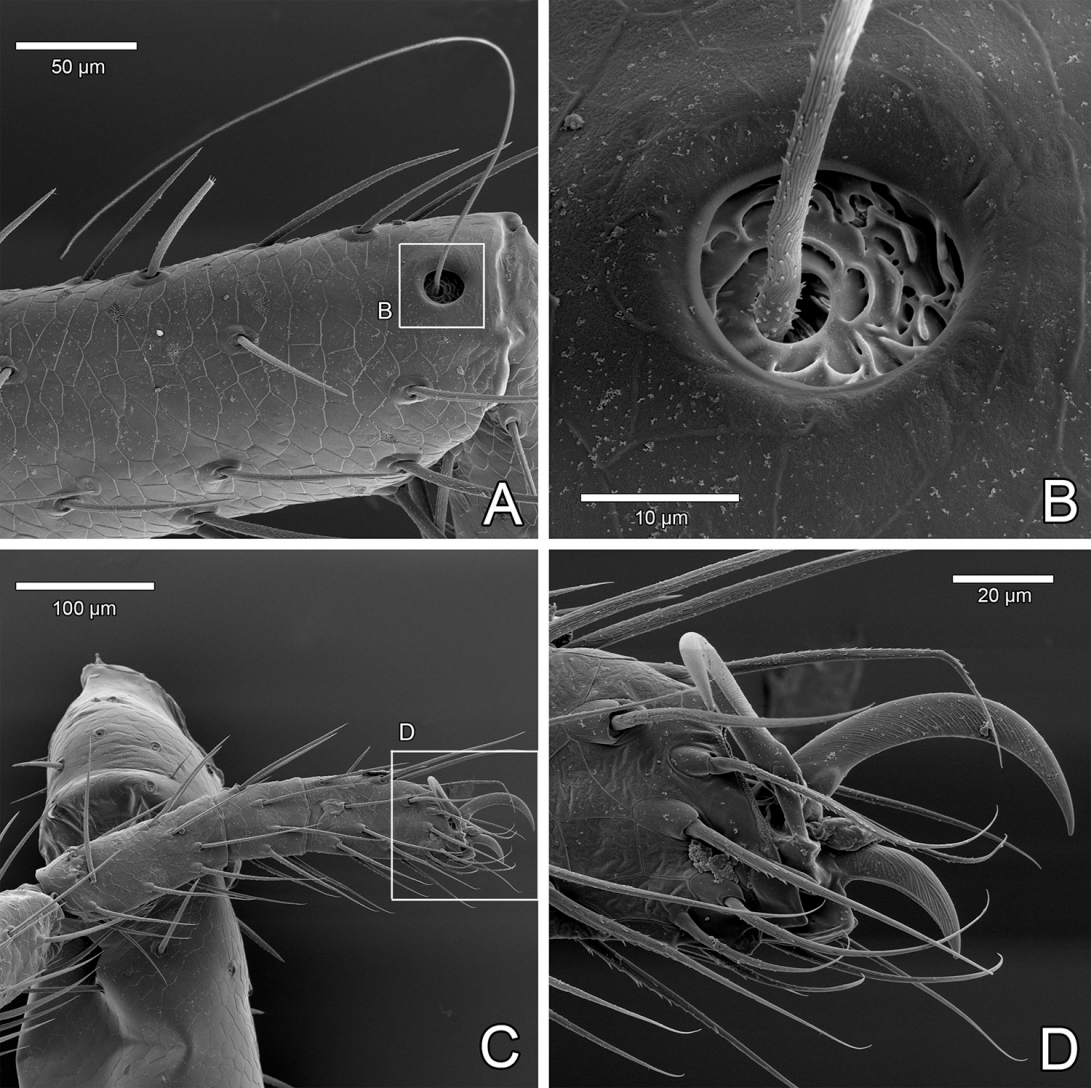
Leg IV of *Surazomus algodoal*. (A) tip of tibia, dorsal view, (B) detail of previous showing trichobothrium base, (C) tip of leg, (D) detail of previous showing tarsal claws.

Tactile leg I has elongated patella and tarsus composed of six tarsomeres (Figs 12 and 13; male holotype with single tarsomere is now confirmed as a malformation, as suspected by Ruiz & Valente [34]). On each tibia I there is a pair of trichobothria (Fig 12A, 12B; unlike tibiae of legs II-IV, which bear a single trichobothrium each; Fig 14A, 14B; [46]). On tarsomeres of leg I there are several club setae (modified, thick setae) interpreted as a special sensorial organ, first noticed by Hansen & Sørensen [18] (Fig 13A–13C, yellow arrows, 13D). Other than these, there are thicker (straight) and more slender/longer setae (that may become hooked in SEM preparation) on the tip of leg I (Fig 13C).

#### Opisthosoma

**Fig 15:**
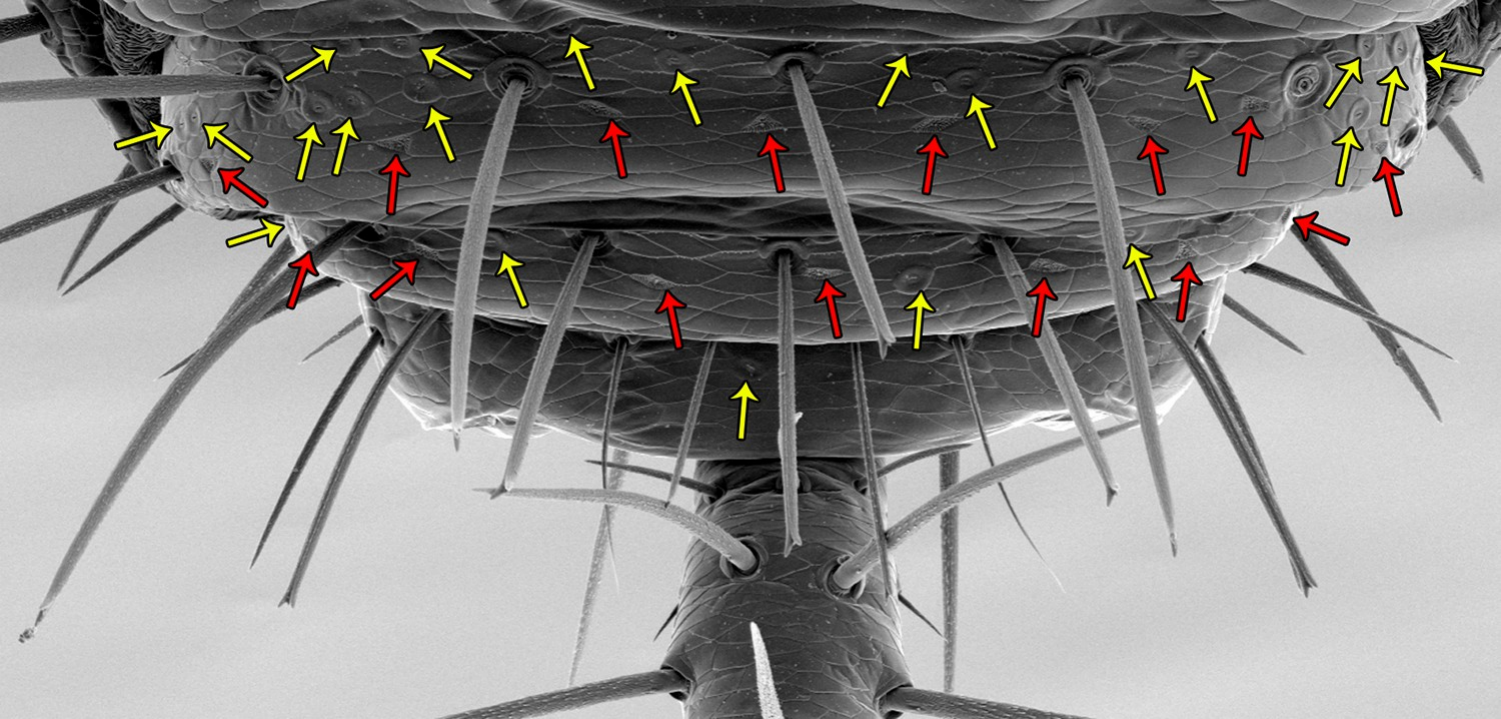
Opisthosoma of *Surazomus algodoal*, female. Ventral view, showing segments X-XII (area with bifid/trifid setae) and base of pygidial flagellum. Yellow arrows: slit-like glandular openings; red arrows: rosette-shaped glandular openings.

Opisthosomal setae vary among individuals. Dorsally: Dm are conservative, Dl1 and Dl2 may be absent; ventrally: Vm2 and Vl1 are conservative, AS between the Vm2 pair and between Vm2 and Vl1 may be absent. Macrosetae become increasingly longer from sternite VIII to end of opisthosoma. Under SEM, ventral macrosetae (Vm/Vl) have bifid/trifid tip, while microsetae (AS) have simple tip. These bifid/trifid macrosetae are present also on ventral portion of the prosoma and are especially elongated in the posterior portion of opisthosoma (Fig 15).

#### Male flagellum (Figs 16–18)

**Fig 16:**
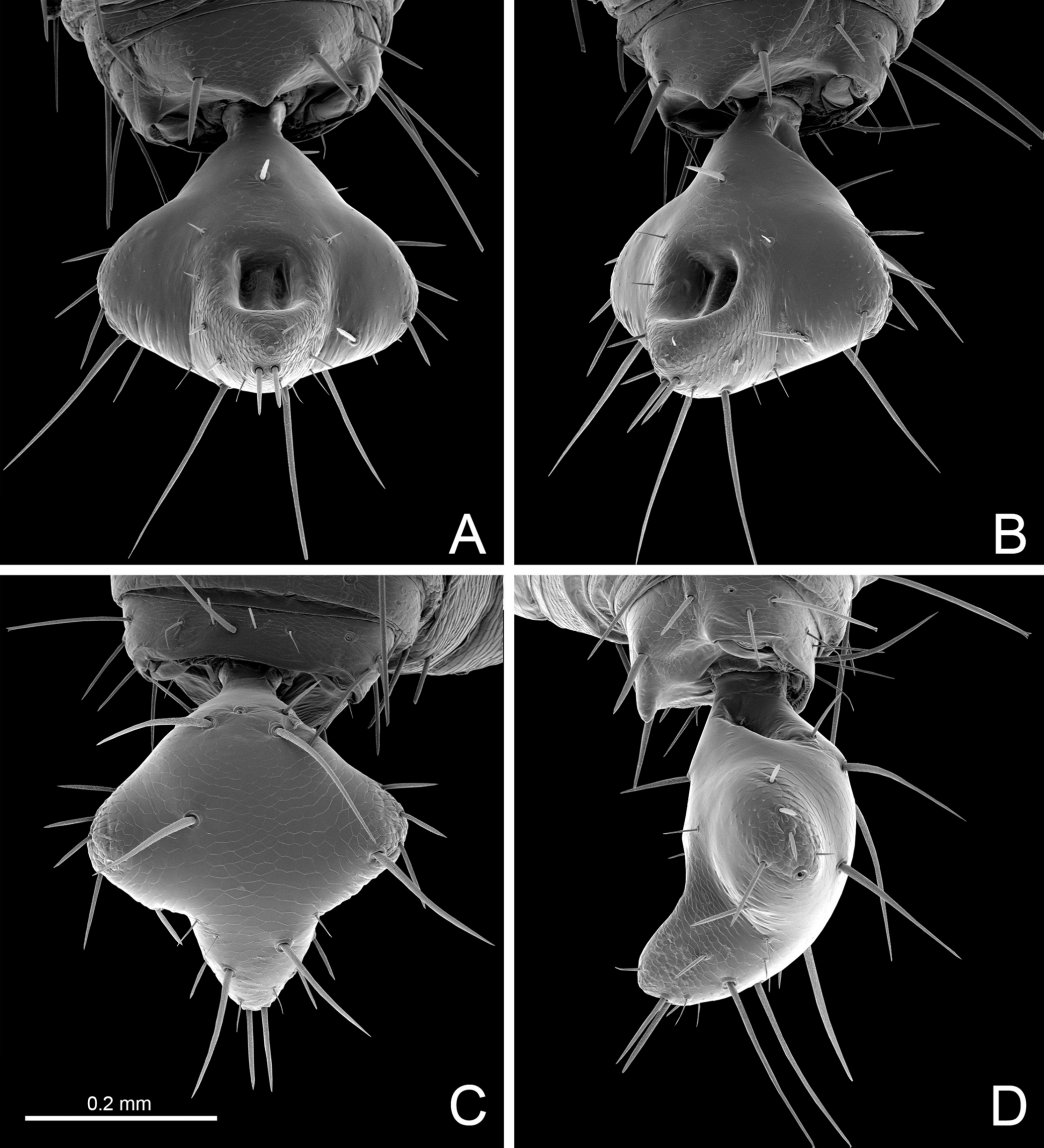
*Surazomus algodoal*, male. (A-D) pygidial flagellum: (A) dorsal view, (B) dorsolateral view, (C) ventral view, (D) lateral view.

**Fig 17:**
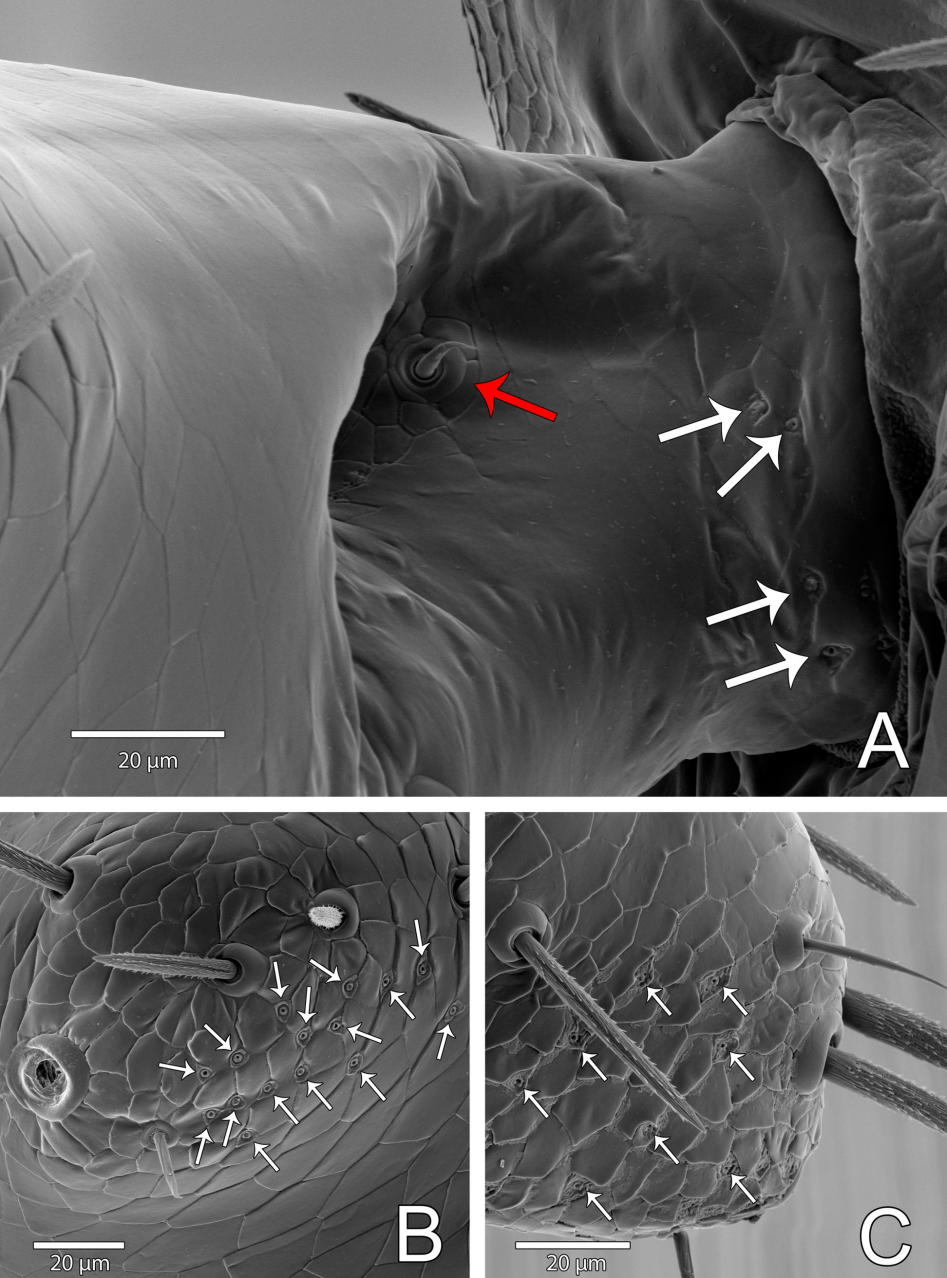
*Surazomus algodoal*, male pygidial flagellum. (A) anterior pocket bearing seta Dl1 (red arrow), (B) lateral lobe bearing several rimmed pores (white arrows); (C) median lobe bearing several rimmed pores (white arrows).

**Fig 18:**
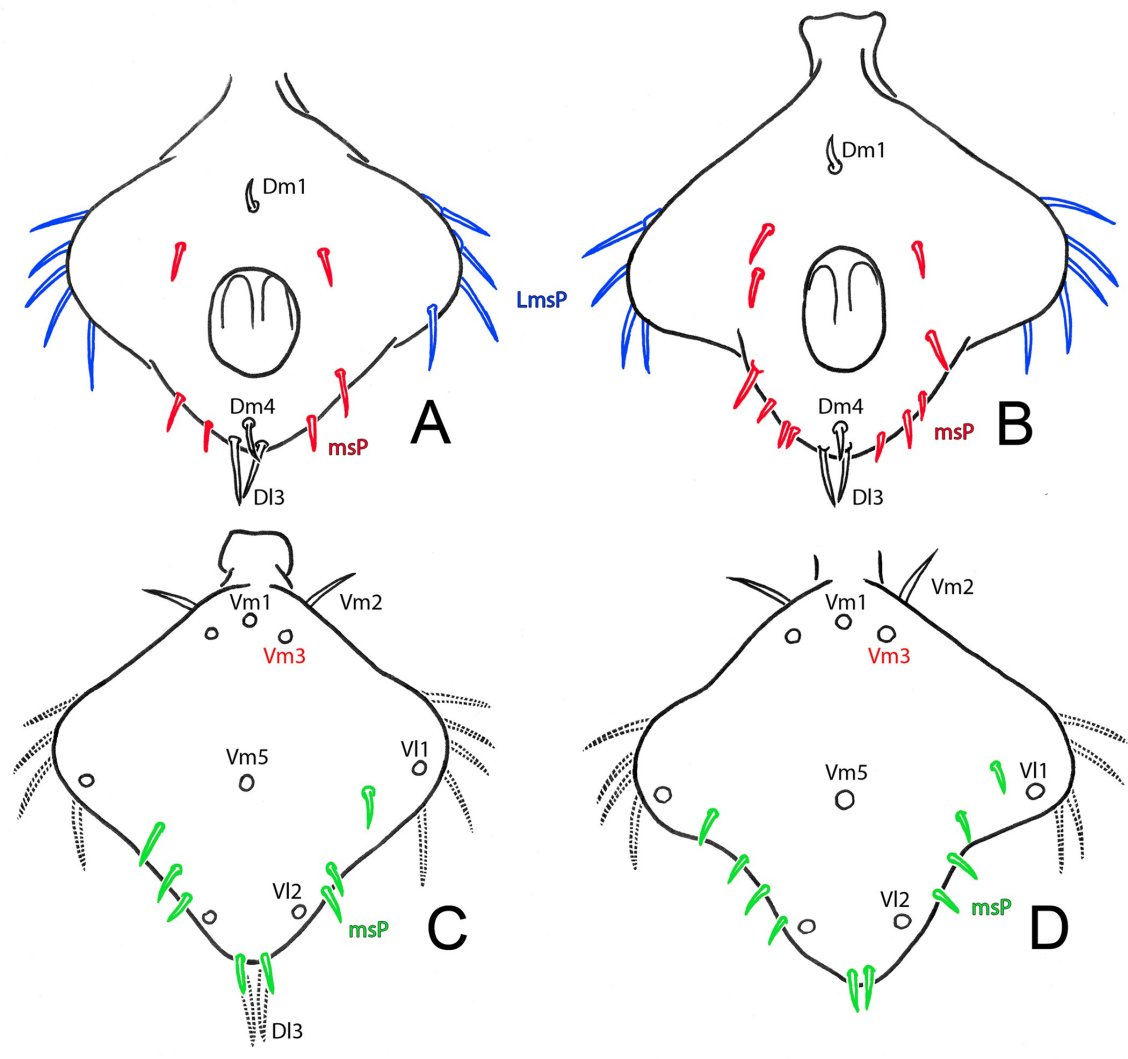
*Surazomus algodoal*, male pygidial flagellum. Variation in microsetae (red for dorsal, blue for lateral and green for ventral setae). (A-B) dorsal view, (C-D) ventral view.

Microsetae Dl1, ignored by Ruiz & Valente [34], but indicated by Ruiz & Valente [3], is corroborated as present, inside AP (anterior pockets; see Ruiz & Valente [3]: figs 8C-D) (Fig 17A, red arrow). All flagellar setae barbed (Fig 17B, 17C). Specimens examined show intraspecific variation in development of lateral lobes (Fig 18). Seta Vm5 may not be centralized (Fig 16C; compare with Fig 18C, 18D); Vl2 pair may not be aligned (Fig 16C; compare with Fig 18C, 18D). Each lateral lobular microseta patch (LmsP) with 4–6 microsetae (Fig 18). Median (posterior) lobe dorsally with 4–6 msP on each side (Fig 18A, 18B), forming an arch that surpasses PP (posterior pocket; see Ruiz & Valente [3]: figs 8C-D) anteriorly. Ventrally with 3–4 microsetae on each side between Vl1 and Vl2 (msP in Fig 18C, 18D; holotype with three setae on each side, not described originally); also, with a pair of microsetae under Dl3 pair (Fig 18C; including the holotype, not described originally).

### Description of other cuticular structures found in *S*. *algodoal*: Sensorial organs and glandular openings

The examination of the ultrastructure of the cuticle of the body of *S*. *algodoal* revealed ten additional types of structures interpreted as sensorial organs and glandular openings, which are described below and summarized in Table 3.

**Table 3:** Classification of cuticular structures in *S*. *algodoal*.

Structure name	Description	Distribution	Function
Slit sensillum (Fig 10H)	As defined by Bertkau [39]	Chelicera, palp (tibia)	Mechanoreceptor (proprioceptor)
Lyriform organ (white arrow in Fig 13B)	As defined by Gaubert [40]	Palp, legs	Mechanoreceptor (proprioceptor)
Trichobothrium (Fig 14A)	As defined by Dahl [38]	Legs (tibia)	Mechanoreceptor (airflow)
Bifid/Trifid tip seta (Fig 15)	Long seta (barbed) with bifid or trifid tip (inconspicuous pore)	Ventral portion of prosoma and opisthosoma, conspicuous on end of opisthosoma	Mechano-thermoreceptor (?)
Tip pore seta (Fig 8D)	Seta bearing a pore on its tip	Chelicera	Chemoreceptor (gustative)
Smooth seta (Fig 13C)	Smooth seta	Tip of leg I	Chemoreceptor (?)
Club seta (Fig 13D)	Short and thick seta, frequently covered with small particles	Tip of leg I	Chemoreceptor (?)
Slit-like glandular opening (Fig 10I)	Limited by a roundish elevation (sometimes divided into two pieces: one around 3/4 of the circumference, and a second closing the circle); in the middle there is a thin membrane bearing an elongated, narrow opening	Palp (tibia), leg I (femur, patella, tibia), posterior end of opisthosoma (ventral)	Glandular
Rosette-shaped glandular opening (Fig 19B)	Described by Santos & Pinto-da-Rocha [21] and Pinto-da-Rocha *et al*. [32] (as glandular pores)	All over the body, rare on carapace, chelicera and leg I (only on tip); concentrated on palp and posterior end of opisthosoma (ventral)	Glandular
Rimmed pore (white arrows in Fig 17B)	Circular pore surrounded by complete thin rim	Male flagellum	Glandular (?)

#### Chelicera

There are sparse slit sensilla on the prolateral surface, as follows: three, near the articulation of the movable finger (prolateral) and a line with 4 on the ventral edge of the chelicera, more basal than G5B setae (white arrows in Fig 8A).

#### Pedipalp

Pedipalp with several sparse rosette-shaped glandular openings, both retrolaterally (Fig 10A, 10B) and prolaterally (white arrows in Fig 10C, 10F, see also Figs 10E, 10G, 11) placed on femur (denser on retrolateral face), patella, tibia and tarsus (the three articles with denser structures on prolateral face), and prolaterally on trochanter. These rosette-shaped glandular openings (Fig 11) were described in detail by Santos & Pinto-da-Rocha [21] and Pinto-da-Rocha *et al*. [32] (named as glandular openings; see discussion below).

Other than the many rosette-shaped glandular openings, the pedipalp has some slit sensilla (Fig 10H, yellow arrows in Fig 10F) on the prolateral face (two on tibia and three on tarsus) and a single slit-like glandular opening on tibia (Fig 10I). The slit-like glandular opening generally occupies the area of a cuticular tile on the surface of the body and are limited by a roundish elevation (sometimes divided into two pieces: one around 3/4 of the circunference, and a second closing the circle); in the middle there is a thin membrane bearing an elongated, narrow opening (Fig 10I). Lyriform organ present dorsally at femur-patella articulation (e.g. green arrow in Fig 10A).

#### Legs

Unlike the walking legs II-IV (Fig 14C, 14D), there is no terminal claw on leg I (Figs 12C, 12I, 13A, 13C). Instead, leg I is modified as a sensorial appendage (see sensorial setae above), but slit sensilla are rare or absent. Lyriform organs are present dorsally near articulations, including between metatarsus and first tarsomere of leg I (Fig 13A, 13B, white arrows). Rosette-shaped glandular openings occur also on the tip of leg I (Fig 13C, red arrows). Long femur I has some slit-like glandular openings along its meso-ventral line, which continue on patella and tibia (Fig 12E–12H).

### Opisthosoma

The posterior ventral portion of the opisthosoma reveals a dense concentration of glandular openings, namely the slit-like (Fig 15, yellow arrows) and the rosette-shaped ones (Fig 15, red arrows).

#### Flagellum

**Fig 19:**
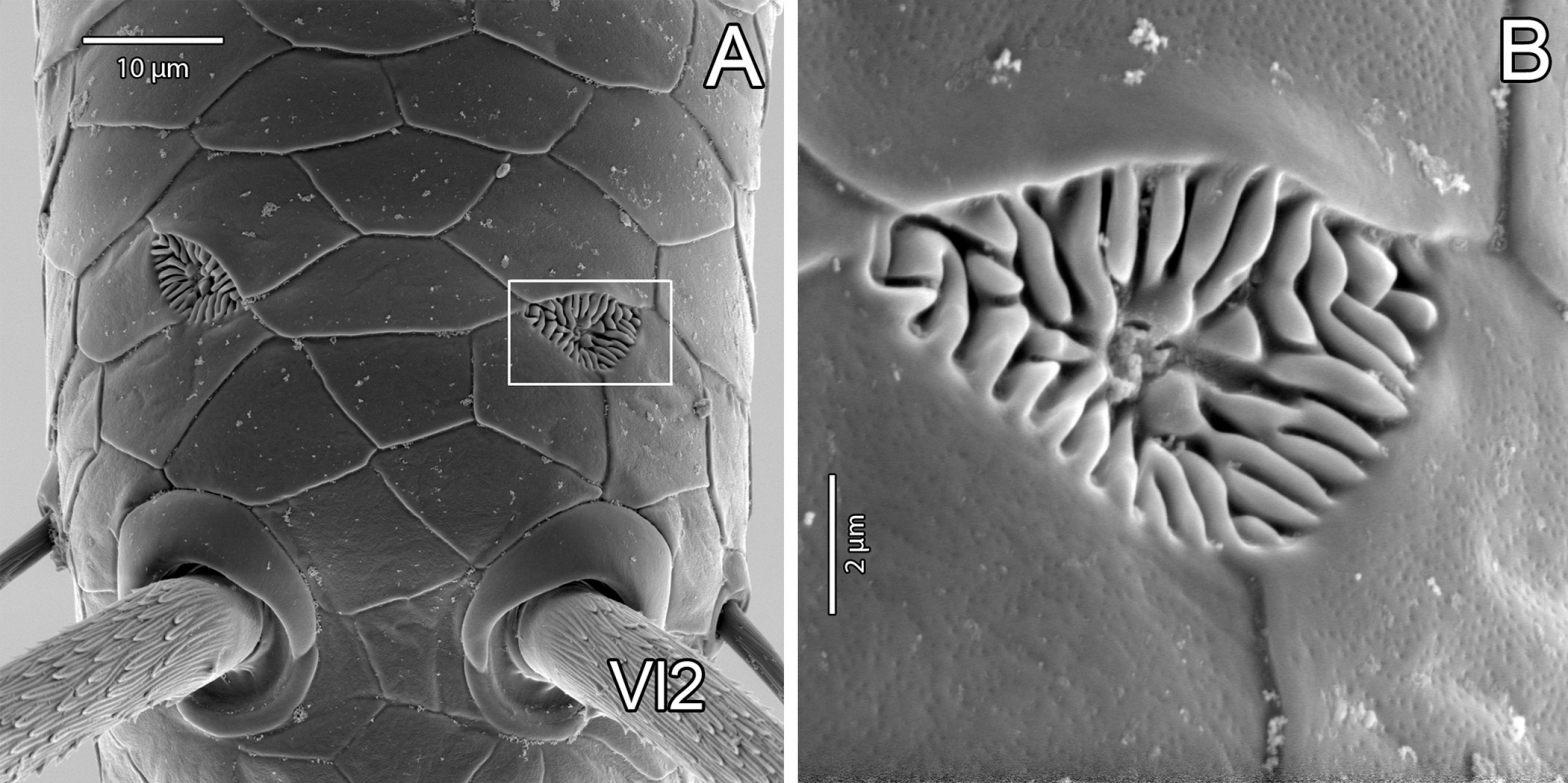
*Surazomus algodoal*, female pygidial flagellum. (A) tip, ventral view, (B) detail of previous illustration showing rosette-shaped glandular opening.

Female with rosette-shaped glandular openings (Fig 19A, 19B) near setae Dm4 and Vl2. Male with sparse rosette-shaped glandular openings between setae Vm5 and Vl2, and with several rimmed pores on lateral and median lobes (Fig 17B, 17C, white arrows). There are also some pores on the base of the male flagellum that may be interpreted as rimmed pores (Fig 17A, white arrows).

### Description of the pygidial gland in *S*. *algodoal*

**Fig 20:**
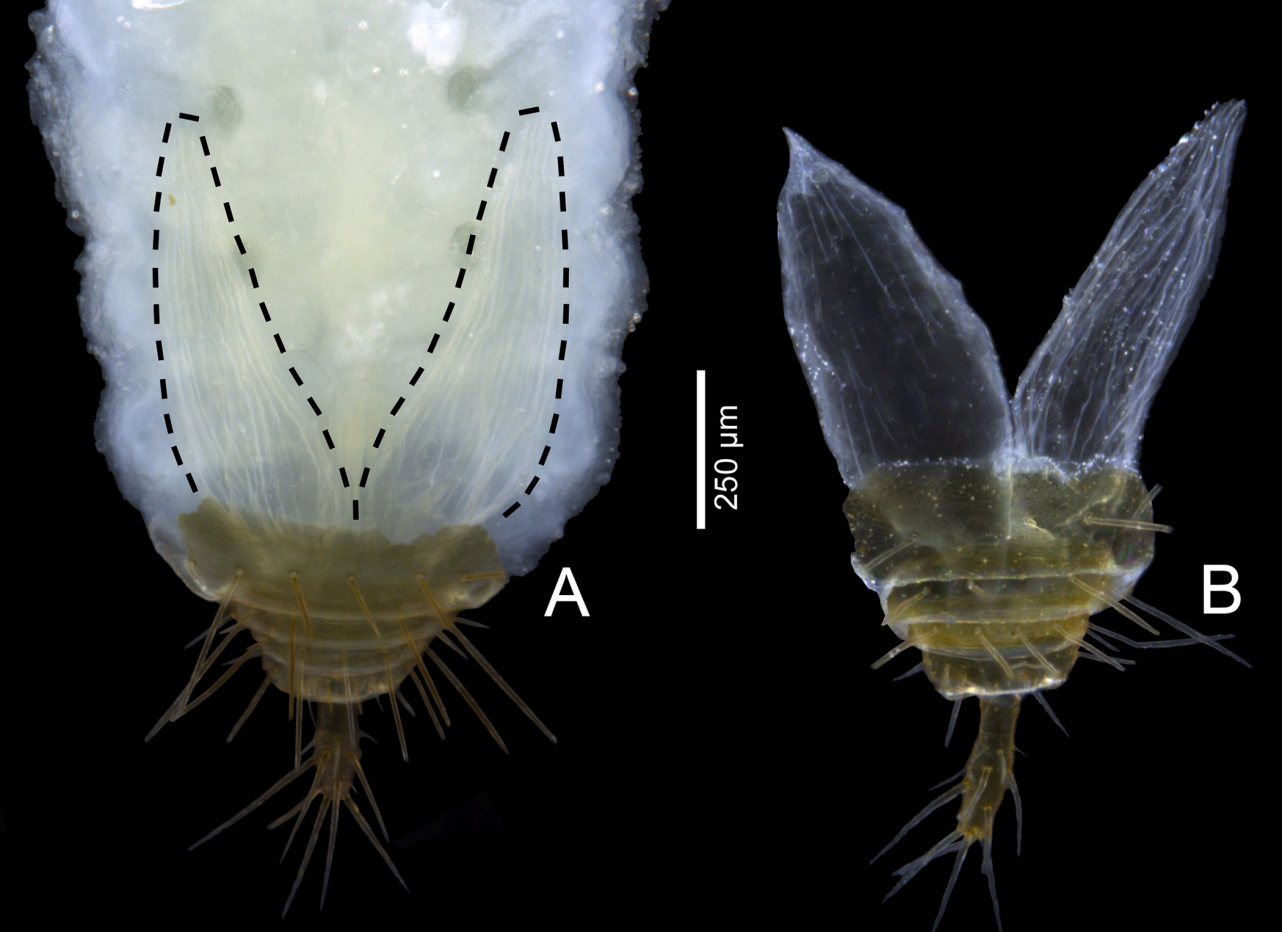
*Surazomus algodoal*, female, tip of opisthosoma. Ventral view (only tergites/sternites IX-XII preserved), showing pygidial glands. (A) before, and (B) after KOH maceration.

**Fig 21:**
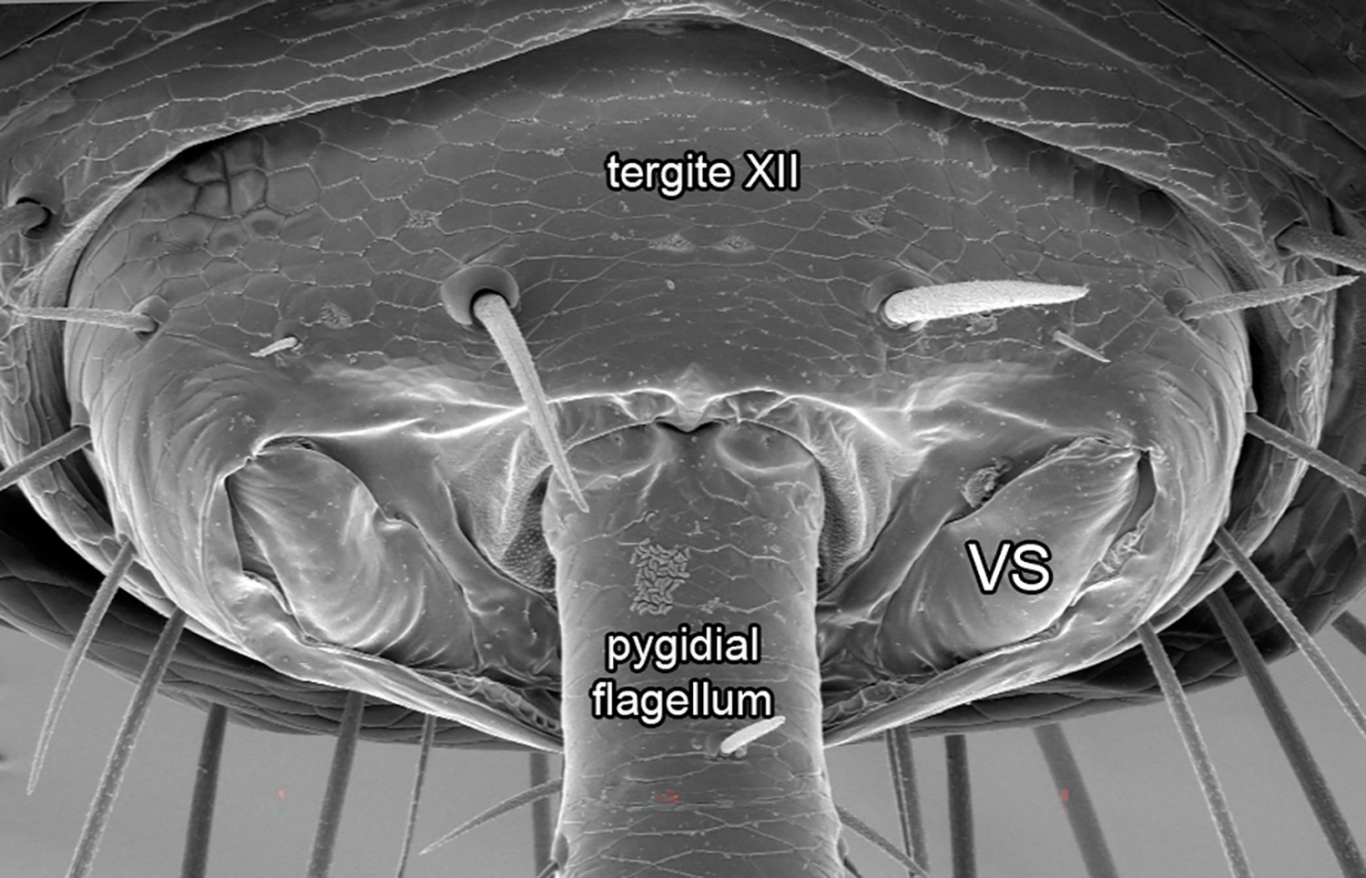
*Surazomus algodoal*, female, tip of opisthosoma. Posterior view, showing vaporizer sclerites of pygidial glands beside the flagellar articulation. Abbreviation: (VS) vaporizer sclerite.

A pair of pygidial glands is present on the posterior portion of the opisthosoma in both sexes, right above the sternites (Fig 20A, 20B). These glands extend from the 6^th^ opisthosomal segment to the posteriormost segment (Fig 2A), opening on each side of the flagellar insertion on opisthosomal segment XII. Unlike in thelyphonids, the openings are covered by a pair of sclerites named herein as the vaporizer sclerites (VS in Figs 2B, 21). These are not movable and the contents of the glands are vaporized through a pair of slits present on the dorsal portion of the complex (Fig 21).

## Discussion

### Sexual dimorphism

In schizomids, the structures used in the mating march were expected to be sexually dimorphic. Despite the chelicerae of the female being used in mating march (e.g. [3]), there is no sexual dimorphism in the chelicera of *S*. *algodoal*. The few G5B setae observed on the female chelicera (three setae in the female; eight in the male; Table 2) may be a variation, since females of other species, such as *Surazomus cuenca* (Rowland & Reddell, 1979) and *Surazomus chiapasensis* seem to have about eight G5B setae [28, 30], as males. That leads us to conclude that the sexual selective pressure may be affecting the counterpart–the male pygidial flagellum. This structure is diverse so much so that it is used in species and group diagnoses [4]. The morphology of the chelicerae may be constrained by its use in predation. Otherwise, the modification on tergite XII forming a posterodorsal process in males (see Ruiz & Valente [34] for more details) certainly is related to female anchoring/stability of the chelicerae during the mating march.

Concerning pedipalp dimorphism, males of *S*. *algodoal* have only slight apophyses on the femur and patella (other species of the genus have larger apophyses, such as *S*. *paitit* Bonaldo & Pinto-da-Rocha, 2007; see Bonaldo & Pinto-da-Rocha [47]: fig 1), and the base of trochanter seta TRv5, named as AP–apical process in other studies (e.g. [23, 24]) is much enlarged–about 1/6 the trochanter length, forming a forceps with the patellar apophysis (Figs 9A, 10E). Based on the current knowledge on the mating march in schizomids (see Ruiz & Valente [3]), there is no plausible explanation for this modification in the male palp regarding male-female interaction. We believe that these structures may be related, instead, to male-male interactions. Such modifications used in male-male agonistic behaviors are common in arachnids, such as in the palps of amblypygids (e.g. [48]) or chelicerae of some spiders (e.g. [49]).

In both sexes of schizomids, leg I is completely altered to a tactile function, while leg IV is modified as a jumping appendage ([50]: 524), with a thick, grasshopper-like femur (Figs 1A, 1B, 2A). Leg I in males is about 1.3x longer than that of the female in *S*. *algodoal* (Fig 1A, 1B), while leg IV is not sexually dimorphic. We do not know whether this is the result of a male-male competition pressure for mating chances (searching for mate and ideal substrate for spermatophore deposition), or a female preference during the complex prenuptial behavior. The male holotype had only the left leg I (see Ruiz & Valente [34]: fig 1) and among the other males more recently collected, a second male also had a single leg I. It is also uncertain how these two males have lost one of their front legs, either in male-male interactions, due to predators, or even during molting.

### Male genitalia

Modder [33] described the male genitalia of short-tailed whip scorpions (*Schizomus crassicaudatus* O. Pickard-Cambridge, 1872) for the first time, but his detailed description was based solely on transverse and sagital cuts of the genital area. Modder [33] divided the genital portion into the dorsal “*vesicula seminalis*” and the ventral “genital chamber”, which are used herein. Modder [33] clearly distinguished the “gonopore”, i.e. the opening from the seminal vesicle into the genital chamber, from the “gonotreme”, the opening that connects the genital chamber to the outside. He also proposed the following terms used herein: “roof” of the genital chamber, extending posteriorly as the “dorsal lip of the gonotreme”; and “floor” of the genital chamber (modifications of the gonosternite near the gonotreme), which extends posteriorly as the “ventral lip of the gonotreme”.

Given his limited capability of reconstructing 3D cuticular structures based on serial cuts, Modder [33] named the several sclerites of the genital chamber as “cuticular lining” (*cut*.) and used letters (*cut*.*a*-*cut*.*h*) to indicate the different structures of the genital chamber. His *cut*.*a* and *cut*.*b* were related to modifications around the gonopore and are not treated in this work. Here we propose an interpretaion of how the 3D structures being described herein match his system of sclerite classification (Table 4).

**Table 4:** New terminology and comparison of male genital structures used herein and by Modder [33] and Giupponi & Kury [37]. * Terms newly proposed.

Name used herein	Modder’s [33] name	Modder’s [33] explanation	Giupponi & Kury [37]
**Gonosternite** *	Floor of genital chamber	-	Genital operculum (GO)
Seminal vesicle	Seminal vesicle	-	-
Genital chamber	Genital chamber	-	-
Gonotreme (Gt)	Gonotreme	-	-
Dorsal lip of gonotreme (DLGt)	Dorsal lip of gonotreme	-	-
Ventral lip of gonotreme (VLGt)	Ventral lip of gonotreme	-	-
**Furcula** (Fu)*	(cuts d and g below)	-	-
**Lateral bar of furcula** (LBFu) *	-	-	-
**Dorsal arm of furcula** (DAFu) *	cut. d	Cuticular reinforcement of gonotreme’s dorsal lip	-
**Ventral arm of furcula** (VAFu) *	cut. g (fig 13)	Cuticular ridge, on floor of genital chamber	-
**Pterapophysis** (Pt) *	cut. g (figs 11 and 12)		-
**Bridge** (Br) *	-	-	-
**Median field** (MF) *	cut. g (fig 10)	-	-
Gonopod (Gp)	-	-	Gonopod
**Fold of gonopod** (FGp) *	-	-	-
Fistula (Fi)	-	-	Fistula (Fi)
**Hook of fistula** (H) *	cut. h	Cuticular reinforcement of gonotreme’s ventral lip	-
*lobus lateralis primus* (LoL1)	-	-	*lobus lateralis primus* (LoL1)
*lobus lateralis secundus* (LoL2)	-	-	*lobus lateralis secundus* (LoL2)
*lamina medialis* (LaM)	-	-	*lamina medialis* (LaM)
**Median apodeme** (MA) *	cut. c	A prominent cuticular process on roof of genital chamber	-
**Lateral flap** (LF) *	cut. e	Cup-like cuticular structure on roof of genital chamber	-
not named herein, but it is the thickened base of the papillate flap	cut. e_2_	(posterior part of cut. e)	-
**Papillate flap** (PF) *	cut. f	Saucer-shaped cuticular structure, bearing papillae, on roof of genital chamber	-
**Median septum** (MS) *	-	-	-
**Anterior Muscles** (AM) *	-	-	-
**Posterior Muscles** (PM) *	-	-	-
**Anterior Transverse Muscles** (ATM) *	-		-
**Posterior Transverse Muscles** (PTM) *	-	-	-
**Oblique Muscles** (OM) *	-	-	-

The morphology of the female genitalia has proven to be of great consistency, both at genus and species levels, and of high value for taxonomic research in schizomids [5]. Although we were able to examine the male genitalia of only a single species in this work, we believe that they might be of equal importance as the female’s.

### Hypotheses of homology for male genital sclerites with other orders

Recent phylogenetic reconstructions based on molecules (e.g. [51]) show that, among the several orders of arachnids, the ones with booklungs (Scorpiones, Araneae, Amblypygi, Thelyphonida and Schizomida) form a natural group (Arachnopulmonata). Except for the scorpions, the other four orders gather into a clade named as Tetrapulmonata, composed of arachnids bearing pairs of booklungs in the 2nd and 3rd opisthosomal segments (pair of the 3rd segment posteriorly lost in schizomids and some spiders). Amblypygi, Thelyphonida and Schizomida are grouped as the Pedipalpi, given that their first leg is used for tactile functions, instead of locomotion. Their palps are also modified into raptorial appendages covered with spines, given the absence of paralyzing toxins in these predators, as opposite to scorpions and spiders. Among the three Pedipalpi orders, Schizomida and Thelyphonida share several modifications and are considered as sister groups, composing the clade Uropygi, long accepted as a monophyletic union. Its synapomorphies include a unique mating march [52], fused palpal coxae (see Ruiz & Valente [3]: fig 2B), tibial trichobothria arranged in a 2-1-1-1, pygidial glands, and elongated patella of leg I [46], among others.

Unlike other tetrapulmonate arachnids, the posterior border of the gonosternite in Schizomida lays hidden over the anterior border of sternite III in males (Figs 4A, 5D). Amblypygids and thelyphonids have large seminal vesicles in the dorsal and anterior portions of the genital area, and the posterior portion of the male genitalia is composed of a large gonopod complex. The gonopods of Amblypygi have been extensively studied (e.g. [37]), and the complex pair of gonopods are wrapped by a malleable sclerite named as the fistula. A similar pair of sclerites, although more sclerotized, is found protecting the ventral face of the gonopods in Thelyphonida, and was also recognized as the fistula (see Seraphim [53]). However, it is still unclear if the amblypygid fistula, a part of the true gonopod, is homologous to Seraphim’s interpretation in thelyphonids, since their fistula is a modification of the border of the ventral lip of the gonotreme, not the base of the gonopod. Seiter *et al*. ([54]: fig 6D) gave a clear illustration in ventral view of the heavily sclerotized structure covering the soft cuticle of the gonopods in the thelyphonid *Mastigoproctus proscorpio* (Latreille, 1806), but named them as “wing-like structures”. Although tempting, it is unclear for us if such heavily sclerotized structures holding the gonopods and the genital chamber of thelyphonids (see also “caudal arches” in Seiter *et al*. [54]: fig 6A) have any homology to the sclerites being described herein for schizomids, especially the furcula. On the other hand, we believe that the most basal portion of the gonopods of Schizomida may be homologous to the true fistula of amblypygids, and hence we use this name. However, this portion of the gonopod in Schizomida has a short, sclerotized hook, which seem to be absent in any other arachnid ever studied.

Amblypygi have very large gonopods, subdivided into multiple lobes (see Giupponi & Kury [37]), while Thelyphonida seem to have simpler gonopods. The simplicity seems to be even more prominent in schizomids, as demonstrated herein. It is still unclear, however, if the simpler forms of thelyponids and schizomids may be a synapomorphy (given they are closely related), or a plesiomorphic feature upon which amblypygids developed largely complex gonopods, which seems more plausible. In this work we tentatively kept the names proposed by Giupponi & Kury [37] for the gonopodal lobes. From the sclerotized fistula, we were able to identify a more basal and simpler lobe as the *lobus lateralis primus*, and a larger and more folded lobe as the *lobus lateralis secundus*, as did those authors. Also, we believe that the third lobe identified in the schizomid’s gonopod could be homologous to Giupponi & Kury’s *lamina medialis*.

Regarding the remaining sclerites of the genital chamber of short-tailed whip scorpions, namely the apodemes and flaps present anteriorly in the dorsal lip, we cannot suggest any homology in the other orders, since the dorsal lip has not been completely understood in other arachnids, despite the rare documentation, such as in Seraphim *et al*. ([53]: fig 4F).

Since this is a first attempt to describe in detail the sclerites of the male genital chamber of a species of schizomids, we expect that our observations may help develop this field and provide a new set of characters for future phylogenetic studies in the order.

### Setae and other cuticular structures

Hubbardiines have rich chelicerae concerning seta types [35]. Length and microtrichia vary greatly, some of them seem to be hollow, some bear a pore on the tip and the base may or not be articulated. The hollow setae have a great potential to be confirmed as sensorial setae, since such morphology is congruent with sensorial setae found in other arthropods [55–57]. Among the hollow ones, setae G3 have a single terminal pore and are not articulated, which suggests a possible gustative function (tasting or revealing palatable prey). Gustative setae in arthropods are hollow and usually have a dendrite extending inside along their length to the pore [56, 58, 59]. Given the position of G3 setae associated to fixed finger of chelicera, a gustative function seems plausible [60–62]. Long setae G1, G2, G5A and G5B are hollow, but their bases are articulated and there seems to be no pore. Such morphology seems to be more congruent with mechanosensorial setae [55, 56, 61], which would make sense, since schizomids need to sense the grasp of the prey between the two fingers of the chelicerae. Seta G6, even if not hollow and with no pores, may also be mechanosensorial, given its position as an antenna at the tip of the chelicera and its developed base. On the other hand, setae G4 and G7, more prolaterally placed, are shorter/stouter and seem not to be hollow or have any pores. Given their position on such an important structure for intake—the chelicera, these setae could still be sensorial [56, 60–62]. The true function of all these setae needs to be confirmed in histological and physiological studies. It is possible that some of these setae also help schizomids groom themselves, since these animals are constantly placing their legs I and palps between the chelicerae (pers. observ.). The row of stalked brush-setae on the movable finger combined with the hyaline teeth (Fig 8C) may also be used for self-cleaning after predation or even cleaning of other body parts taken to the chelicerae. Grooming is an important event in arachnid behavior, including schizomids [63], which can prevent the establishment and development of microorganisms on the cuticle, such as fungus spores, as shown in *S*. *algodoal* (Fig 8A–8C), or as already reported on chelicerae of other hubbardiines (*Basidiobolus* Eidam, 1886 fungus in Giupponi *et al*. [29]: fig 3C; unnoticed spore in Villarreal & García [22]: fig 7; probably in Delgado-Santa & Armas [64]: fig 3B, 3C). The form of the brush-setae of the movable finger seems congruent with grooming (see Engel [65]). This function could also be confirmed with observation of live specimens, mainly after ingesting prey. Despite the diversity of setae and other structures in the chelicera of a single species, their contribution to phylogenetic reconstruction within Hubbardiines seems of limited use, since all the seta types seem to be present and similar in number and form throughout the subfamily (e.g. [28]). However, these structures may serve as a morphological marker for the group.

The guard tooth of the movable finger of the chelicera seems to be pressed against the (bifid) tooth of the fixed finger (Fig 9C). This could be an adaptation to hold smaller preying body parts. Still regarding predation, the observation under SEM showed us that the setae present along a meso-ventral stripe on palpal patella (Pm), tibia (Ti, Tm and Tv) and tarsus (TAi and TAm) with brushes on distal halves may act as a single organ, possibly increasing grasp when holding and manipulating prey body parts. The multiple microtrichia present on the prolateral side of such setae seem to enlarge greatly the adherence between the seta and prey body, as demonstrated in the spider claw tuft [66, 67], which enables spiders to climb on smooth surfaces. Another comparison may be made with the scopula on prolateral side of front leg in the spider-predator spider genus *Palpimanus* Dufour, 1820 (Palpimanidae; see Pekár *et al*. [68]). The widening of the cheliceral G1 setae could also help the palps in this task. G1 setae are wide in the distal half and bear a field of microtrichia facing retrolaterally (see Villarreal *et al*. [27]: fig 34; Monjaraz-Ruedas & Francke [23]: fig 42), which certainly increase prey grasp.

Regarding setae of the pygidial flagellum, the female flagellum of *S*. *algodoal* has the same seta pattern described for hubbardiines [24]. Hence, these structures are not expected to contribute to phylogenetic studies, given the conservative pattern among hubbardiines. Male macrosetae, on the other hand, seem to be good markers in phylogenetic reconstructions, since they have variable features across the subfamily, along the shape of the male flagellum. Our results show that male flagellar microsetae are more variable within a single species than macrosetae, rendering microsetae of limited use in comparative studies.

Slit sensilla, lyriform organs and trichobothria are traditional mechanoreceptors present in most arachnids [61, 69–71]. Trichobothria have been documented in several arachnids, including Schizomida (e.g. [18, 46]). The pattern found in *S*. *algodoal* follows that of other short-tailed whipscorpions, with a 2-1-1-1 trichobothria on leg tibiae. This pattern is considered a synapomorphy shared by Schizomida and Thelyphonida [46]. Slit sensilla and lyriform organs, on the other hand, although being recorded as present in most arachnids (e.g. [46]), have not been mapped on most orders. After Hansen & Sørensen [18], these structures have not been largely studied in schizomids, but the 3 slit pattern on the distal chelicera near fang articulation can be seen in many illustrations available in the literature for hubbardiines (e.g. [5]: fig 13; [21]: fig 9; [27]: fig 30; [23]: fig 99; [29]: fig 3C; [28]: fig 13) and protoschizomids [72]: fig 10). We have also found slit sensilla prolaterally on the palpal tibia. The slit sensilla on the chelicera and palp of *S*. *algodoal* may sense deformation or strain on the cuticle generated during predation, since these appendages seem both to act as pray graspers. We expect that the mapping of such sensorial organs on the different species may contribute to phylogenetic reconstruction in many arachnid groups, including schizomids.

The bifid/trifid macrosetae have been documented (but ignored) on the ventral portion of the opisthosomal segments 10–12 of both sexes at least in *Rowlandius* Reddell & Cokendolpher, 1995 ([31]: fig 5A; [29]: figs 2B, 6D), *Naderiore* Pinto-da-Rocha, Andrade & Moreno-González, 2016 and *Cangazomus* Pinto-da-Rocha, Andrade & Moreno-González, 2016 ([32]: figs 1, 5, 7, 9), and *Stenochrus* Chamberlin, 1922 ([36]: fig 4C). However, to our knowledge, these structures had not been interpreted as sensorial organs so far. There is no obvious pore on its tip, but the characteristic morphology is very similar to some “no pore” (np) sensorial setae found in ticks (np/C: setae dl2 e laI1 on tarsi I; see Hess & Loftus [73]; Hess & Vlimant [74]: fig 5), in which they were confirmed as mechano- and thermoreceptors. Histological studies may help solve the properties of this seta type in schizomids.

Smooth and club setae are restricted to the tip of sensorial leg I. Hence, we suggest that their sensorial nature is plausible. Although these setae have been documented in SEM illustrations of previous studies (e.g. [21]), they received no special treatment and we still do not understand their function in schizomid life. We suggest that both the smooth and the club setae may have hygroreceptor function, since they are no-pore setae and they are present on the antenniform leg [60–62, 69, 75–77]. Given that terrestrial arthropods need to monitor the presence/abundance of water in the environment, several different hygrorecetors have evolved, most of them placed anteriorly on the body of arthropods (e.g. the Tömösváry organ in Myriapoda, tarsal organ in spider leg I, tarsal sensillum on leg I of harvestmen). Since schizomids have no pit- (as typically present in spiders) or a pore-bearing (harvestmen) tarsal organ, it is possible that at least one of these structures (smooth or club setae) may be related to such a function.

Schizomids have three types of pores on their cuticles that were suggested to be glandular openings. Two of those are well distributed throughout their bodies, while the third type is restricted to the male flagellum. Of the two well distributed glandular openings, one is called the rosette-shaped glandular opening (see Seiter *et al*. [41]; previously “glandular opening/pore” by Santos & Pinto-da-Rocha [21] and Pinto-da-Rocha *et al*. [32]; “uropygid pore” by Santos *et al*. [31]), and the other is called the slit-like glandular opening (see Seiter *et al*. [41]) (Table 3).

The rosette-shaped glandular openings in schizomids consist of a simple pore surrounded by a large area covered with radiating grooves (Fig 11B). A homologous structure seems to be present in thelyphonids: a simple pore flanked by a pair or set of papillae, but with a smaller area covered with radiating grooves (see Santos & Pinto-da-Rocha [21]: figs 17–19). The slit-like glandular openings are present in schizomids and thelyphonids and have similar morphology (Fig 10I; see Seiter *et al*. [41]: fig 5f).

Wolff *et al*. [78, 79] also found two types of glandular openings well distributed on the body of whip spiders (Amblypygi). The first, named as the major glandular opening, consists of a pore surrounded by two valves, sometimes itself surrounded by a series of radiating structures (see Wolff *et al*. [78]: figs 2, 5F; [79]: figs 7B, 8; see also Hebets & Chapman [80]: fig 3c, 3e, 3f; Seiter *et al*. [41]: fig 9; Seiter *et al*. [81]: fig 9). The second, named as the minor glandular opening, is a simple pore with no valves (see Wolff *et al*. [78]: fig 5F; [79]: fig 7D). According to Wolff *et al*. [78], these glands are distributed in close proximity to each other and the products of the minor and the major gland openings would self-assemble to compose a super hydrophobic cerotegument in amblypygids (see Seiter *et al*. [81]: fig 1). Seiter *et al*. [41] assumed that the two-valved major glandular opening described by Wolff *et al*. [78] would be homologous to the slit-like glandular openings of schizomids and thelyphonids, given their overall slit-like morphology. However, since some species of Amblypygi have radiating structures around the major glandular opening (see Wolff *et al*. [79]; Seiter *et al*. [81]), Seiter *et al*. [41] also hypothetized that the major glandular opening of amblypygids could, at the same time, be homologous to the rosette-shaped glandular openings of schizomids and thelyphonids. No homology hypothesis was proposed by them between the amblypygid minor glandular opening and other arachnid orders.

We suggest that our interpretation of glandular types is more parsimonious than that of Seiter *et al*. [41]: the two glandular types of Uropygi (schizomids + thelyphonids) may be homologous to the two glandular types of Amblypygi, i.e., the uropygid rosette-shaped and the amblypygid major glandular openings would be homologous, while the uropygid slit-like glandular opening would be homologous to the amblypygid minor glandular opening, regardless of their detailed morphology and function. Under our interpretation, the two-gland system would be a synapomorphy of Pedipalpi. Santos & Pinto-da-Rocha [21] proposed that the uropygid rosette-like glandular opening could be compared with structures of amblypygids, comprising a synapomorphy for Pedipalpi, but that comparison seems mistaken, since the proposal of homology was established between the rosette with the pit- and the plate-organs of amblypygids, which are sensorial organs present on leg I. The major/minor glandular system had not been described yet. These homologies and putative synapomorphy still need to be tested in a phylogenetic analysis.

Despite the fact that the two-gland system may be present in all Pedipalpi, their function in schizomids is still not understood. As far as we know, the products of the two gland types combine to create a hydrophobic cerotegument (amorphous epicuticular secretion coat in most thelyphonids and granular micropatterned cerotegument in amblypygids and at least in one species of thelyphonid; see Seiter *et al*. [41]). Schizomida, however, appear as having smooth cuticles composed of a tile pattern in all known electron micrographs (as in Fig 11). Although both slit-like and rosette-shaped glandular openings are densely present ventrally on the end of the opisthosoma, which could indicate an association between their products, the distribution of such structures on the palps and legs does not seem congruent (e.g. rosette-shaped glandular openings on palps and not on leg I, slit-like glandular openings rare on palps but abundant on leg I). It is still unclear whether the epicuticular tegument has always been removed prior to the SEM images (possibly diluted in ethanol?) or if schizomids have indeed lost the cerotegument [41]. If present, legs and palps could act together to ease the assemblage of the products of the two glands, by rubbing their glandular openings over the body after molting.

The third type of glandular opening, the rimmed pore, as mentioned above, is restricted to the male flagellum of schizomids. They were first recorded below Vl1 setae of the male flagellum of *Rowlandius pedrosoi* Giupponi, Miranda & Villarreal, 2016, treated simply as “glands” ([29]: fig 2F), and of *Naderiore carajas* Pinto-da-Rocha, Andrade & Moreno-González, 2016 and *Cangazomus xikrin* Pinto-da-Rocha, Andrade & Moreno-González, 2016, treated as non-radiating gland pores ([32]: fig 13). These structures are present only in adult males and are interpreted by us as openings of sexual glands, possibly related to the mating march. The shape of the male flagellum of schizomids has recently been reviewed [4], but these glands have not received attention. Since the presence of rimmed pores may explain the development of lateral lobes in adult male flagella, we argue that it is worth describing them whenever possible.

### Pygidial glands

Our results clearly show the presence of well-developed pygidial glands in *S*. *algodoal*. Whether these structures are present in all species of schizomids is still an open question, but very likely. They are present in the sister-group (Thelyphonida) and paired openings of opisthosomal defensive glands (on either side of the anus) have been coded as present in both extant families of Schizomida (represented by the genera *Stenochrus* and *Protoschizomus* Rowland, 1975) ([82]: char. 46; [83]: char. 86; [84]: char. 122; [46]: char. 102). Their use in defense is not doubtful ([50]: 524), but such behavior has not been observed in *S*. *algodoal*.
